# miR‐21 sustains CD28 signalling and low‐affinity T‐cell responses at the expense of self‐tolerance

**DOI:** 10.1002/cti2.1321

**Published:** 2021-09-21

**Authors:** Maya Fedeli, Mirela Kuka, Annamaria Finardi, Francesca Albano, Valentina Viganò, Matteo Iannacone, Roberto Furlan, Paolo Dellabona, Giulia Casorati

**Affiliations:** ^1^ Experimental Immunology Unit Division of Immunology, Transplantation, and Infectious Diseases IRCCS San Raffaele Scientific Institute Milan Italy; ^2^ Vita‐Salute San Raffaele University Milan Italy; ^3^ Dynamics of Immune Responses Unit Division of Immunology, Transplantation, and Infectious Diseases IRCCS San Raffaele Scientific Institute Milan Italy; ^4^ Clinical Neuroimmunology Unit Institute of Experimental Neurology IRCCS San Raffaele Scientific Institute Milan Italy; ^5^ Experimental Imaging Centre IRCCS San Raffaele Scientific Institute Milan Italy

**Keywords:** autoimmunity, CD28, costimulation, iNKT cells, miR‐21, T cells

## Abstract

**Objective:**

miR‐21 is highly expressed in iNKT and activated T cells, but its T‐cell autonomous functions are poorly defined. We sought to investigate the role of miR‐21 in the development and functions of T and iNKT cells, representing adaptive and innate‐like populations, respectively.

**Methods:**

We studied mice with a conditional deletion of miR‐21 in all mature T lymphocytes.

**Results:**

Thymic and peripheral T and iNKT compartments were normal in miR‐21 KO mice. Upon activation *in vitro*, miR‐21 depletion reduced T‐cell survival, T_H_17 polarisation and, remarkably, T‐ and iNKT cell ability to respond to low‐affinity antigens, without altering their response to high‐affinity ones. Mechanistically, miR‐21 sustained CD28‐dependent costimulation pathways required to lower the T‐cell activation threshold, inhibiting its repressors in a positive feedback circuit, in turn increasing T‐cell sensitivity to antigenic stimulation and survival. Upon immunisation with the low‐affinity self‐epitope MOG_35–55_, miR‐21 KO mice were indeed less susceptible than WT animals to the induction of experimental autoimmune encephalomyelitis, whereas they mounted normal T‐cell responses against high‐affinity viral epitopes generated upon lymphocytic choriomeningitis virus infection.

**Conclusion:**

The induction of T‐cell responses to weak antigens (signal 1) depends on CD28 costimulation (signal 2). miR‐21 sustains CD28 costimulation, decreasing the T‐cell activation threshold and increasing their sensitivity to antigenic stimulation and survival, broadening the immune surveillance range. This occurs at the cost of unleashing autoimmunity, resulting from the recognition of weak self‐antigens by autoreactive immune responses. Thus, miR‐21 fine‐tunes T‐cell response and self‐/non‐self‐discrimination.

## Graphical abstract

Low‐affinity antigens triggering weak TCR signalling (Signal 1) require CD28 costimulation (signal 2) to elicit T‐cell responses. In this study, we found that miR‐21 sustains the CD28 costimulation pathway, via inhibition of its repressors in a positive feedback circuit, decreasing the T‐cell activation threshold. This broadens the range of detected antigens, at the cost of unleashing autoimmunity resulting from the recognition of weak self‐antigens by autoreactive T cells. Thus, miR‐21 fine‐tunes T‐cell response and self/non‐self‐discrimination.

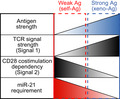

## Introduction

miRNAs are small non‐coding RNAs. miRNAs modulate the expression of target mRNAs via a sequence‐specific hybridisation to their 3′‐untranslated region, resulting either in the block of translation or in the degradation of their targets. miRNAs are conserved across species and play a critical role in several biological processes, such as development, cell differentiation, cell‐cycle control, metabolism and immune response. miRNA expression or function is significantly modified in many disease states, including cancer, heart failure and viral infections. miR‐21 is considered one of the ‘oncomiRs’, because of its elevated expression in many tumors^1^ and its oncogenic function, predominantly through the inhibition of cellular apoptosis,^2^ via targeting three oncosuppressor genes involved in apoptosis and proliferation: phosphatase and tensin homologue (PTEN),^3^ programmed cell death 4 (PDCD4)^4^ and large tumor suppressor kinase 1 (LATS1).^5^ miR‐21 targets also Sprouty (SPRY) family members that mediate receptor tyrosine kinase signalling and MAP kinase (MAPK) pathway in response to growth factors, and promotes ERK and JNK signalling in activated T cells by inhibiting SPRY‐1, an inhibitor of ERK and JNK.^6^ miR‐21 is more expressed by memory than naïve T cells^7^ and is upregulated in activated CD4^+^ and CD8^+^ T cells,^8^, ^9^, ^10^ in turn regulating T‐cell effector response, apoptosis, proliferation, and migration depending on the differentiation status of the T cells.^11^, ^12^ miR‐21 is also upregulated in Tregs, compared with conventional CD4^+^ T cells,^13^ supporting a functional role in activation‐experienced T cells. Yet, what is the function of miR‐21 in T cells independent of its expression in other relevant cells of the immune system is incompletely understood.

Invariant natural killer T cells (iNKT cells) are a unique population of innate‐like T lymphocytes characterised by the expression of a semi‐invariant αβ TCR, formed in mice by an invariant Vα14‐Jα18 chain paired with few distinctive Vβ chains.^14^ This semi‐invariant TCR is is specific for lipid antigens presented by the MHC class I‐related molecule CD1d.^14^ iNKT cells pass through an agonist selection process by which they acquire an activated/effector phenotype already at the thymic level, which occurs in several steps: stage 0 (CD24^+^CD44^low^NK1.1^−^); stage 1 (CD24^−^CD44^low^NK1.1^−^); stage 2 (CD44^hi^NK1.1^−^); and stage 3 (CD44^hi^NK1.1^+^).^14^ Through this process, iNKT cells gain distinct T_H_1 (iNKT1), T_H_2 (iNKT2) and T_H_17 (iNKT17) effector phenotypes, regulated by the master transcription factors T‐bet, GATA3, PLZF and RORγt, respectively.^15^ This thymic iNKT cell developmental programme critically depends on the RNase III enzymes Dicer and Drosha that generate functional microRNAs, and on miR‐150, miR‐155, miR‐181ab, Let‐7 and miR‐17~92 or miR‐183‐96‐182 family clusters^16^, ^17^, ^18^, ^19^, ^20^, ^21^, ^22^, ^23^ that regulate different iNKT cell development steps in a time‐dependent manner, and are distinct from their effects on T cells.^17^, ^18^, ^19^, ^20^, ^21^, ^22^, ^24^ Furthermore, among the 17 miRNAs that are differentially expressed between thymic iNKT and T cells, miR‐21 was the only one overexpressed in iNKT cells.^16^

Given the relevance of miR‐21 for the cellular immune response, and the poor definition of its T‐cell autonomous activity, we sought to investigate the role of miR‐21 in iNKT and T‐cell development and function, taking advantage of mice with a conditional deletion of the miR‐21‐containing gene starting from the immature double‐positive (DP) stage of thymocyte development, but with a normal miR‐21 expression in any other immune cells.

## Results

### miR‐21 is upregulated in both T and iNKT cells upon activation

**Figure 1:**
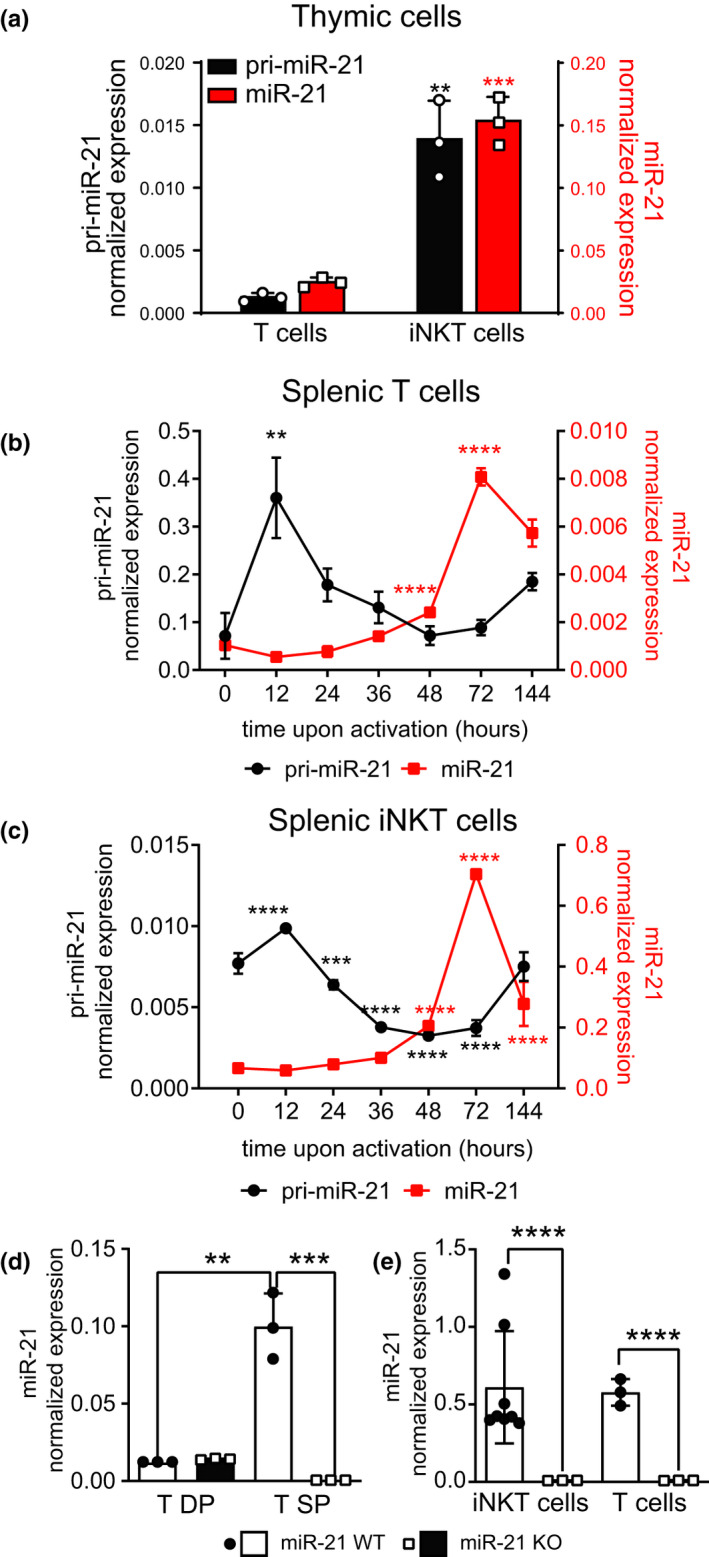
miR‐21 is highly expressed by iNKT cells and is upregulated upon activation. miR‐21 and pri‐miR‐21 were measured in parallel in the indicated cells. **(a)** Sorted thymic mature T cells (TCR‐β^+^ tetrCD1d^−^) and iNKT cells (TCR‐β^+^ tetrCD1d^+^) from C57BL/6N mice. **(b)** Splenic T cells from C57BL/6N mice activated with αCD3/αCD28 beads for the indicated times. **(c)** Splenic iNKT cells purified from iVa14Tg mice activated with αCD3/αCD28 beads for the indicated times. **(d, e)** miR‐21 levels were analysed in cells sorted from miR‐21 KO (miR‐21^fl/fl^ × pCD4‐Cre^+/−^) and littermate WT controls (miR‐21^fl/fl^ × pCD4‐Cre^−/−^) in the indicated cells: **(d)** DP (HSA^high^CD4^+^CD8^+^) and SP (pooled HSA^low^CD4^+^CD8^−^ or HSA^low^CD4^−^CD8^+^ cells) thymocytes; **(e)** hepatic iNKT (CD19^−^MHCI^ab−^TCR‐β^+^tetrCD1d^+^) and T cells (CD19^−^MHCI^ab−^TCR‐β^+^tetrCD1d^−^). Data are representative of 2 or 3 experiments with 3 or 4 mice per experiment. Data in the histograms represent mean ± SD. **P ≤ 0.01, ***P ≤ 0.001 by ANOVA and ****P ≤ 0.0001 by ANOVA.

We previously showed that thymic iNKT cells express more miR‐21 than T cells.^16^ To confirm this result, we isolated thymic iNKT and T cells and compared their expression levels of either mature miR‐21 or pri‐miR‐21, the primary transcript generated by RNA polII that is first cleaved by the RNase Drosha, to generate a precursor miRNA (pre‐miRNA) that is processed by Dicer into mature miRNAs.^25^ As shown in Figure 1a, thymic iNKT cells expressed higher levels of pri‐miR‐21 than T cells, suggesting increased transcription, which was paralleled by greater mature miR‐21 expression. As miR‐21 expression is upregulated in T cells upon TCR‐dependent stimulation, we sought to compare the dynamic induction of pri‐ and mature miR‐21 in peripheral T and iNKT cells upon activation *ex vivo*. T and iNKT cells were purified form the spleen of WT C57BL/6N and iVa14 Tg mice, respectively, and activated *in vitro* with αCD3/αCD28 beads for the indicated times to dynamically assess the induction of the miRNAs at different times from activation. In both cell types, activation upregulated pri‐miR‐21 followed by the mature miR‐21 increase with similar kinetics, reaching its maximum by 72 h post‐stimulation (Figure 1b and c) and supporting a transcriptional induction of miR‐21 expression upon TCR engagement.

### miR‐21 KO iNKT and T cells do not show any gross developmental defect

The above results suggested a possible role for miR‐21 in controlling development and effector differentiation of T and iNKT cells, which was hence investigated in mice in which the floxed miR‐21‐containing gene, Tmem49,^26^ was deleted by CD4‐Cre in thymocytes at the immature DP stage, resulting in the loss of the miRNA in all T and iNKT cells. A qRT‐PCR performed on sorted thymic DP (HSA^high^CD4^+^CD8^+^) and SP (pooled HSA^low^CD4^+^CD8^−^ and HSA^low^CD4^−^CD8^+^ cells) thymocytes (Figure 1d), and on hepatic T and iNKT cells (Figure 1e) confirmed the effective miR‐21 deletion in the T‐cell lineage of miR‐21 KO mice (miR‐21^fl/fl^ × pCD4‐Cre^+^) compared with littermate WT controls (miR‐21^fl/fl^ × pCD4‐Cre^−^).

The analysis of iNKT and T‐cell thymic development in 4‐ or 8‐week (wk)‐old miR‐21 KO mice did not show any gross defect (Supplementary figure 1). At both time points, thymocyte number was comparable in miR‐21 KO and WT mice. iNKT cell frequency, number and phenotype at all maturation stage (0‐1‐2‐3) were comparable in KO and WT animals (Supplementary figure 1a–g). Furthermore, in 8‐week‐old mice the partitioning of iNKT cells into the NKT1, NKT2 and NKT17 effector subsets was similar in miR‐21 KO and WT mice (Supplementary figure 1h). Hence, these data showed that the deletion of miR‐21 at DP stage did not influence iNKT cell thymic development. iNKT cell frequency, numbers and effector subset distribution were not significantly different between miR‐21 KO and WT mice also in the periphery (Supplementary figure 2a, c, e), with the expected NKT1 and NKT17 enrichment in the spleen and LNs, respectively (Supplementary figure 2b, d, f). We next assessed the ability of miR‐21 KO iNKT cell to help B‐cell response to protein antigens.^27^ miR‐21 and WT mice were immunised with protein antigens mixed with either α‐GalCer, a strong CD1d‐restricted iNKT cell agonist, or Alum as adjuvants (Supplementary figure 3). Both in spleen (Supplementary figure 3a, b) and in LN (Supplementary figure 3c, d) of both miR‐21 KO and WT mice, α‐GalCer, but not Alum, induced the differentiation of iNKT_FH_ cells able to help B cells (Supplementary figure 3a, c), whereas both α‐GalCer and Alum induced the differentiation of CD4^+^ T_FH_ cells (Supplementary figure 3b, d), all as expected.^27^ Consistent with normal induction of both iNKT_FH_ and T_FH_ cells, also Ag‐specific IgM and IgG titres rose and persisted comparably in both miR‐21 KO and WT animals, including the secondary IgG increase upon a rechallenge with the Ag alone without adjuvant (Supplementary figure 3e, f).

Also, peripheral frequency and number of T cells were comparable in miR‐21 WT and KO mice in terms of CD4/CD8 coreceptor usage, effector subset distribution (naïve CD44^low^CD62L^hi^; central memory CD44^hi^CD62L^hi^; effector memory CD44^hi^CD62L^low^) (Supplementary table 1 and Supplementary figure 4). Furthermore, although miR‐21 is more expressed in Tregs than in conventional CD4^+^ T cells,^13^, ^28^ natural Tregs normally developed in blood, spleen and LN in miR‐21 KO mice (Supplementary figure 5a–c).

### T_H_17 polarisation and CD4^+^ T‐cell survival are reduced without miR‐21

We next wondered whether miR‐21 KO iNKT and T cells retained their abilities for effector differentiation and cytokine production, since miR‐21 is upregulated upon activation (Figure 1b and c). iNKT cell production of cytokines (IL‐4 and IFN‐γ) following the injection of the strong agonist α‐GalCer *in vivo* did not show difference between miR‐21 KO and WT animals, either by quantifying the two cytokines in the serum (Supplementary figure 6a) or by intracellular staining (Supplementary figure 6b). No differences between miR‐21 KO and WT animals were also detected in the percentage of iNKT cells from spleen, liver and LNs producing IL‐4 or IFN‐γ upon activation *in vitro* (Supplementary figure 6c–f). Similarly, miR‐21 KO splenic CD4^+^ and CD8^+^ T cells produced normal amounts of IL‐4 and IFN‐γ upon activation with PMA/ionomycin *in vitro* (Supplementary figure 7).

**Figure 2:**
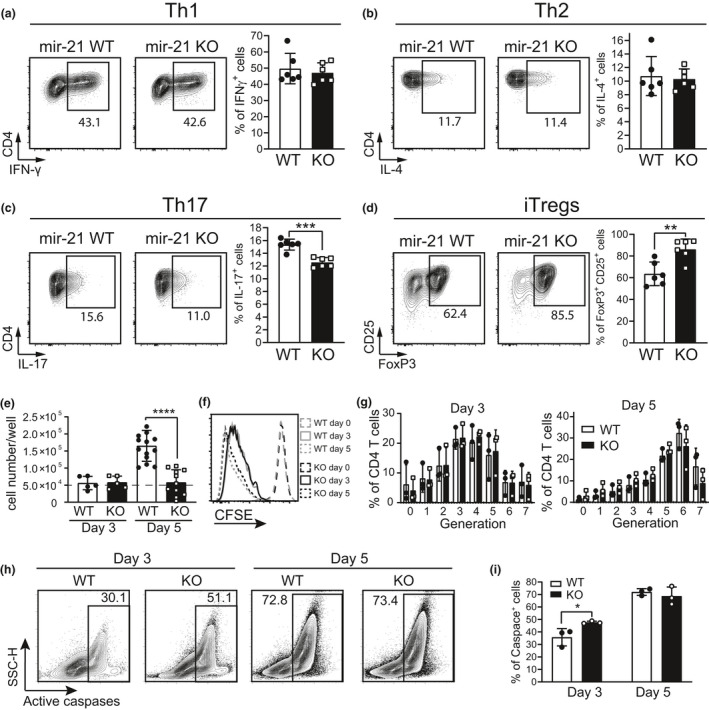
CD4 T helper cells develop correctly but die early of apoptosis even in not polarising conditions. Naïve CD4 T cells, purified from miR‐21 WT and KO spleens and LN, were cultured *in vitro* in polarising conditions: **(a)** T_H_1, **(b)** T_H_2, **(c)** T_H_17 and **(d)** iTregs. Cells after 4 days were then screened for cytokine production: **(a)** IFN‐γ, **(b)** IL‐4, **(c)** IL‐17 or **(d)** FoxP3/CD25 expression. Cells in **a–d** are gated on viable TCRβ^+^ CD4^+^ cells. **(e)** Cell counts of purified naïve CD4 T cells kept in culture for 3 or 5 days stimulated with anti‐CD3 and anti‐CD28 only. The dashed line represents the initial number of cells plated at day 0. **(f)** Representative plot of the proliferation of cells analysed in **e** assessed by labelling with CFSE. **(g)** Graphs of the generations counted by CFSE labelling at day 3 and at day 5 of T_H_0 culture. **(h)** Representative staining for apoptotic cells in cultures as in **e**. **(i)** Graph of apoptotic cells found in cultures after 3 or 5 days. Data are representative of 4 experiments with 3–6 mice per experiment. Data in the histograms represent mean ± SD, statistical significance was tested by a two‐tailed unpaired *t*‐test **(a**–**e**, **i)** and by ANOVA **(g)** (*P ≤ 0.05, **P ≤ 0.01, ***P ≤ 0.001 and ****P ≤ 0.0001).

We next wondered whether the effector plasticity of CD4^+^ T cells was preserved in the absence of miR‐21. Purified naïve CD4^+^ T cells were stimulated *in vitro* to acquire T_H_1, T_H_2 or T_H_17 effector functions (Figure 2a–c, respectively). T_H_1 and T_H_2 differentiated normally into the expected phenotypes, as revealed by their capability to produce their signature cytokines IFN‐γ or IL‐4; however, IL‐17‐producing cells were instead significantly reduced in miR‐21 KO mice compared with WT mice (Figure 2c). We also differentiated naïve CD4 T cells into inducible T regulatory cells (iTregs), and we found that miR‐21 KO cells were more prone to acquire this phenotype (Figure 2d). Furthermore, we noticed that T cells purified from miR‐21 KO mice did not persist in culture, even in neutral T_H_0 conditions when activated by anti‐CD3 plus anti‐CD28 mAbs, and their counts drastically decreased after 5 days of culture (Figure 2e), whereas WT cells had almost triplicated. We asked whether this cell loss was because of a reduced proliferation or to an increased death. miR‐21 KO T cells proliferated normally *in vitro*, with a slight but not significant decrease in the percentage of T cells reaching the last generations at day 5 compared with WT T cells (Figure 2f and g). By contrast, the frequency of miR‐21 KO T cells dying at day +3 of activation *in vitro* was significantly higher than that of WT T cells (Figure 2h and i), coinciding with the maximal upregulation of miR‐21 (Figure 1b), in turn resulting in the reduced number of recovered miR‐21 KO T cells at day +5 of culture (Figure 2e). These results were also validated by Annexin V/DAPI staining (Supplementary figure 8).

Collectively, these results revealed that miR‐21 deletion from all T cells resulted in a: (1) selective, although modest, impairment of T_H_17 differentiation; (2) increased ability to become iTregs; and (3) decreased T‐cell survival upon activation *in vitro* with optimal polyclonal stimulation, whereas the functionality of the peripheral iNKT cell compartment was essentially unaffected by the lack of miR‐21 in response to the strong α‐GalCer stimulus.

### miR‐21 depletion impairs iNKT and T‐cell response to low‐affinity antigens

**Figure 3:**
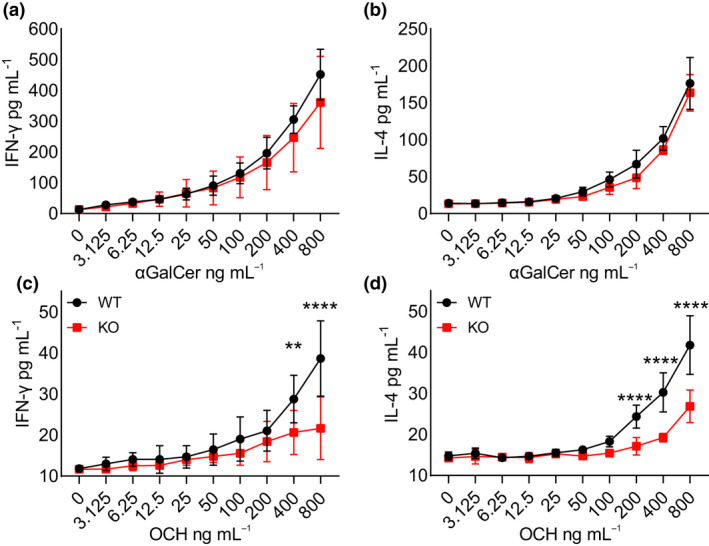
miR‐21 KO iNKT cells fail to respond to low‐affinity lipidic antigens. *In vitro* production of IFN‐γ and IL‐4, assessed by ELISA on supernatants of a 24‐h culture of *ex vivo* splenocytes containing 5000 iNKT cells (as determined by FACS checking of iNKT cell percentage for each mouse), challenged with increasing doses of **(a, b)** α‐GalCer or **(c, d)** OCH. NA, not activated; data are representative of 3 experiments with 3 or 4 mice per experiment. Data represent mean ± SD, statistical significance was tested by 2‐way ANOVA (**P ≤ 0.01 and ****P ≤ 0.0001).

The above results were obtained by challenging T cells with strong activation signals, yet miRNAs are known to work as fine tuner of cellular reaction. Hence, we wondered whether iNKT and T‐cell responses towards low‐affinity antigens that deliver limiting signalling were also preserved in the absence of miR‐21 expression. We first compared the response of iNKT cells against α‐GalCer and OCH, because the affinity of the iTCR for the CD1d‐OCH complex is approximately 20% of that for CD1d‐α‐GalCer one, making OCH a low‐affinity candidate Ag.^29^ Equal number of splenic miR‐21 KO or WT iNKT cells was stimulated *in vitro* with increasing concentration of the two lipids, and iNKT cell activation was assessed by determining the secretion of IFN‐γ and IL‐4 in the culture supernatant. As shown in Figure 3, whereas the response to α‐GalCer was comparable between miR‐21 KO and WT iNKT cells (Figure 3a and b), that to OCH was significantly reduced in miR‐21 KO compared with the WT cells (Figure 3c and d). Thus, these results suggested that miR‐21 facilitates iNKT cell response to low‐affinity lipid antigens.

**Figure 4:**
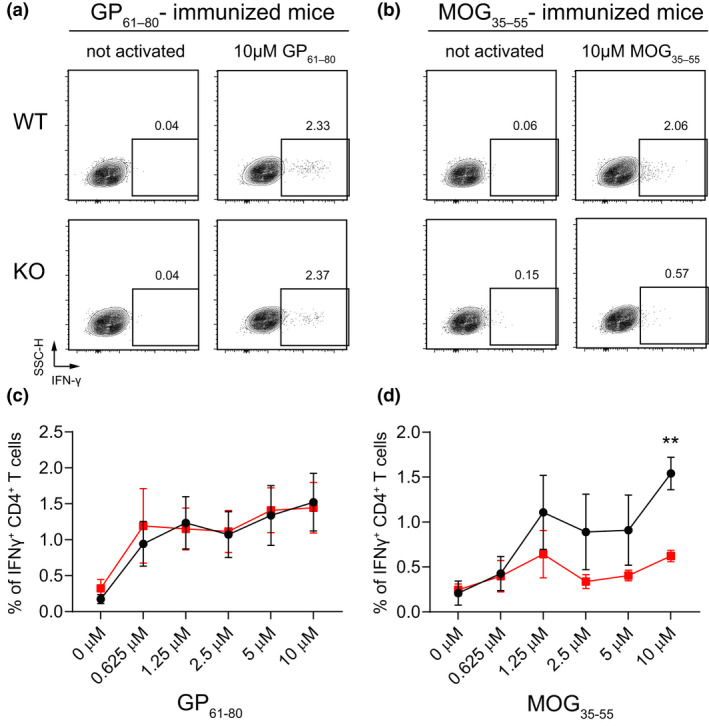
miR‐21 KO T cells fail to respond to low‐affinity peptides. miR‐21 WT and KO mice were immunised with 200 μg of MOG_35–55_ or GP_61–80_ in CFA. After 10 days, mice were sacrificed and 2 × 10^6^ splenocytes were restimulated *in vitro* for 5 h with increasing concentrations of the respective peptide. IFN‐γ production was assessed by intracellular staining. **(a, b)** Representative plots for WT and KO **(a)** GP_61–80_‐ and **(b)** MOG_35–55_‐immunised mice. **(c, d)** Graphs for **(c)** GP_61–80_‐ and **(d)** MOG_35–55_‐immunised animals. Data are representative of 2 experiments with 3 or 4 mice per group per experiment. Data represent mean ± SD, statistical significance was tested by 2‐way ANOVA (**P ≤ 0.01).

We next investigated the need for miR‐21 also in the response of T cells to low‐affinity peptide epitopes. For that, we compared two well‐known models of low‐ and high‐affinity CD4^+^ T‐cell epitopes, respectively, namely MOG_35–55_ and GP_61–80_.^30^, ^31^ MOG_35–55_ is a low‐affinity T‐cell epitope derived from the self‐protein myelin oligodendrocyte glycoprotein that elicits experimental autoimmune encephalomyelitis (EAE) upon immunisation of mice. By contrast, GP_61–80_ is a high‐affinity T‐cell epitope derived from the murine lymphocytic choriomeningitis virus (LCMV). The mean affinity of polyclonal MOG_35–55_‐specific CD4^+^ T cells is 26‐fold lower than that of GP_61–80_‐specific CD4^+^ T cells, and immunisation of WT C57BL/6 mice with MOG_35–55_ results in a greater frequency of tetramer‐negative low‐affinity T cells, as compared to the immunisation with GP_61–80_.^30^, ^31^ WT and miR‐21 KO mice were hence immunised with one of the two peptides, and their splenic T cells were compared at single cell level, by intracellular staining, for IFN‐γ production upon restimulation *in vitro* with increasing concentration of the respective peptide (Figure 4). Immunisation of miR‐21 KO and WT mice with the strong peptide epitope GP_61–80_ elicited a comparable frequency of CD4^+^ T cells producing IFN‐γ (Figure 4c). By contrast, immunisation with the low‐affinity MOG_35–55_ peptide epitope induced substantially less IFN‐γ‐producing CD4^+^ T cells in miR‐21 KO mice compared with the WT ones (Figure 4d).

Together, these results showed that miR‐21 facilitates also the T‐cell response to low‐affinity peptide epitopes.

### miR‐21 sustains CD28‐dependent costimulation supporting weak TCR signals

**Figure 5:**
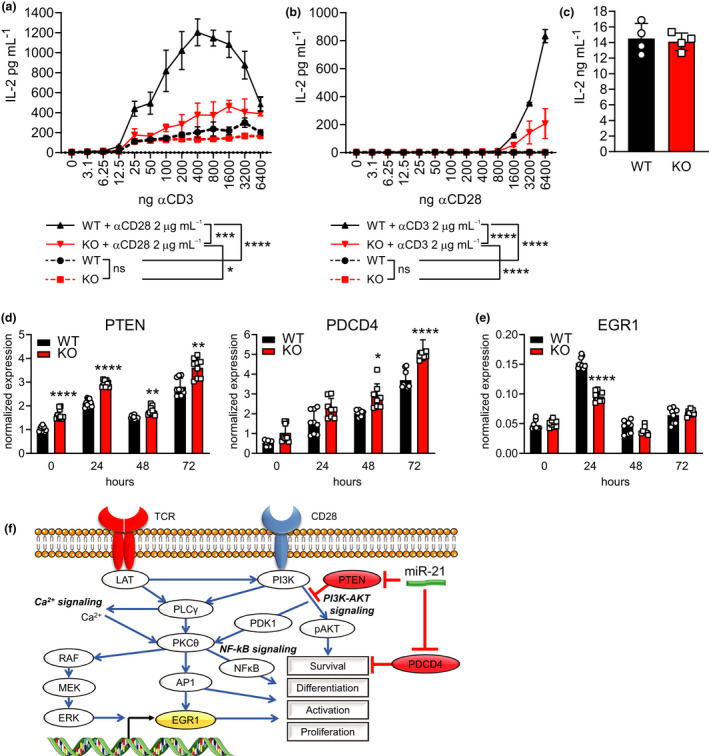
miR‐21 sustains CD28‐dependent costimulation through its targets. **(a, b)** CD4^+^ T cells from miR‐21 WT or KO mice were activated *in vitro* by increasing amounts of insoluble **(a)** anti‐CD3 mAb or **(b)** anti‐CD28 mAb, in the presence or in the absence of 2 μg mL^−1^ anti‐CD28 mAb or anti‐CD3 mAb, respectively, or **(c)** in the presence of 25 ng mL^−1^ PMA plus 1 μg mL^−1^ ionomycin. IL‐2 release was assessed by ELISA on supernatants after 24 h. **(d, e)** qRT‐PCR of **(d)** miR‐21 targets PTEN and PDCD4 or **(e)** EGR1, after *in vitro* activation of miR‐21 WT and KO CD4^+^ T cells with αCD3/αCD28 beads for the indicated times. **(f)** Schematic representation of miR‐21 contribution to CD28 costimulation and response to TCR engagement in T cells. One of 2 or 3 comparable experiments, each performed with 3 or 4 mice per group, is shown. Data in the histograms represent mean ± SD. *P ≤ 0.05, **P ≤ 0.01, ***P ≤ 0.001 and ****P ≤ 0.0001 by a *t*‐test in **(c)** and by 2‐way ANOVA in **(d, e)**; for the graph in **a** and **b**, the areas under the curves were compared.

So far, we have shown that miR‐21 depletion in T cells results in a reduced survival, effector differentiation and response to weak antigen stimulation, but not to strong agonists. These functions are typically evoked in T cells by CD28‐dependent costimulation. T‐cell activation relies on TCR‐dependent signal 1 combined with CD28‐dependent costimulatory signal 2.^32^ Costimulatory signals lower the activation threshold of T cells making them more sensitive to antigenic stimulation, as well as sustain their proliferation, effector differentiation and survival. We thus reasoned that miR‐21 may facilitate T‐cell response to low‐affinity antigens, and support their cytokine production and survival, by sustaining CD28‐dependent costimulatory signals. CD4^+^ T cells from miR‐21‐deficient or miR‐21‐proficient mice were hence activated *in vitro* in two ways: (1) by increasing concentrations of insoluble anti‐CD3 mAb, to mimic increasing signal 1, in the presence or in the absence of an optimal concentration of anti‐CD28 mAb to deliver costimulatory signal 2; and (2) by titrating anti‐CD28 mAb in the presence of a fixed suboptimal concentration of insoluble anti‐CD3 mAb. T‐cell activation was assessed by measuring the release of IL‐2, whose production is highly costimulation‐dependent. Both miR‐21‐deficient and miR‐21‐proficient CD4^+^ T cells responded equally to signal 1 alone, implying that the TCR signalling was unaffected by the lack of miR‐21 expression (Figure 5a). By contrast, the addition of the CD28‐dependent costimulation sustained the IL‐2 production at low TCR‐dependent stimulation only in WT CD4^+^ T cells, and not in miR‐21 KO ones, supporting a defective costimulatory signalling resulting from the lack of miR‐21 expression (Figure 5a). Consistent with this result, miR‐21 KO CD4 T cells responded also significantly less than WT cells to anti‐CD28 mAb titration (Figure 5b) in the presence of fixed suboptimal anti‐CD3 mAb. As a further control for optimal and stimulation, WT and miR‐21 KO CD4 T cells were activated *in vitro* with PMA/ionomycin (Figure 5c), which bypass the membrane proximal components of the TCR‐CD28 signalling pathway components^33^ registering no differences between them and confirming the previous *ex vivo* results obtained via ICS (Supplementary figure 7).

Next, we sought to assess the molecular bases subtending the activation, effector differentiation and survival defects of miR‐21 KO CD4^+^ T cells. PTEN and PDCD4 are two validated miR‐21 direct targets that are involved in the attenuation of the CD28‐dependent PI3K signalling and AKT‐dependent cell survival, respectively.^3^, ^4^ The lack of miR‐21 would result in unphysiological increase in PTEN and PDCD4 expression and suppressive functions on CD28‐dependent signalling and survival. The time course quantification of PTEN and PDCD4 transcripts (Figure 5d) in the miR‐21 KO or WT CD4^+^ T cells *ex vivo* and upon activation with optimal anti‐CD3+CD28 stimulation showed that PTEN was constitutively overexpressed in the miR‐21 KO cells and significantly upregulated upon activation, compared with WT cells. However, PDCD4 underwent significant induction in the absence of miR‐21 expression, suggesting an increased inhibition of costimulatory and survival signals by either molecule. Furthermore, although not a direct target of miR‐21, the expression of early growth response gene 1 (EGR1) is rapidly elevated in T cells upon TCR engagement^34^ and in response to CD28‐dependent costimulation^35^ and PI3K signalling,^36^ suggesting its detection as a proxy to assess the integrity of the CD28‐dependent costimulatory pathway. Consistent with attenuated CD28‐dependent costimulatory signalling in the absence of miR‐21 expression, EGR1 induction was significantly less intense in miR‐21‐deficient than in miR‐21‐proficient CD4^+^ T cells at early time following TCR+CD28 stimulation (Figure 5e).

Collectively, these results support a role for miR‐21 in sustaining T‐cell activation, effector differentiation and survival by fine‐tuning the CD28‐dependent signalling pathway (Figure 5f) via restriction of suppressor molecules, following a positive feedback mechanism typical of miRNA action.

### The EAE penetrance is reduced in miR‐21 KO mice

**Figure 6:**
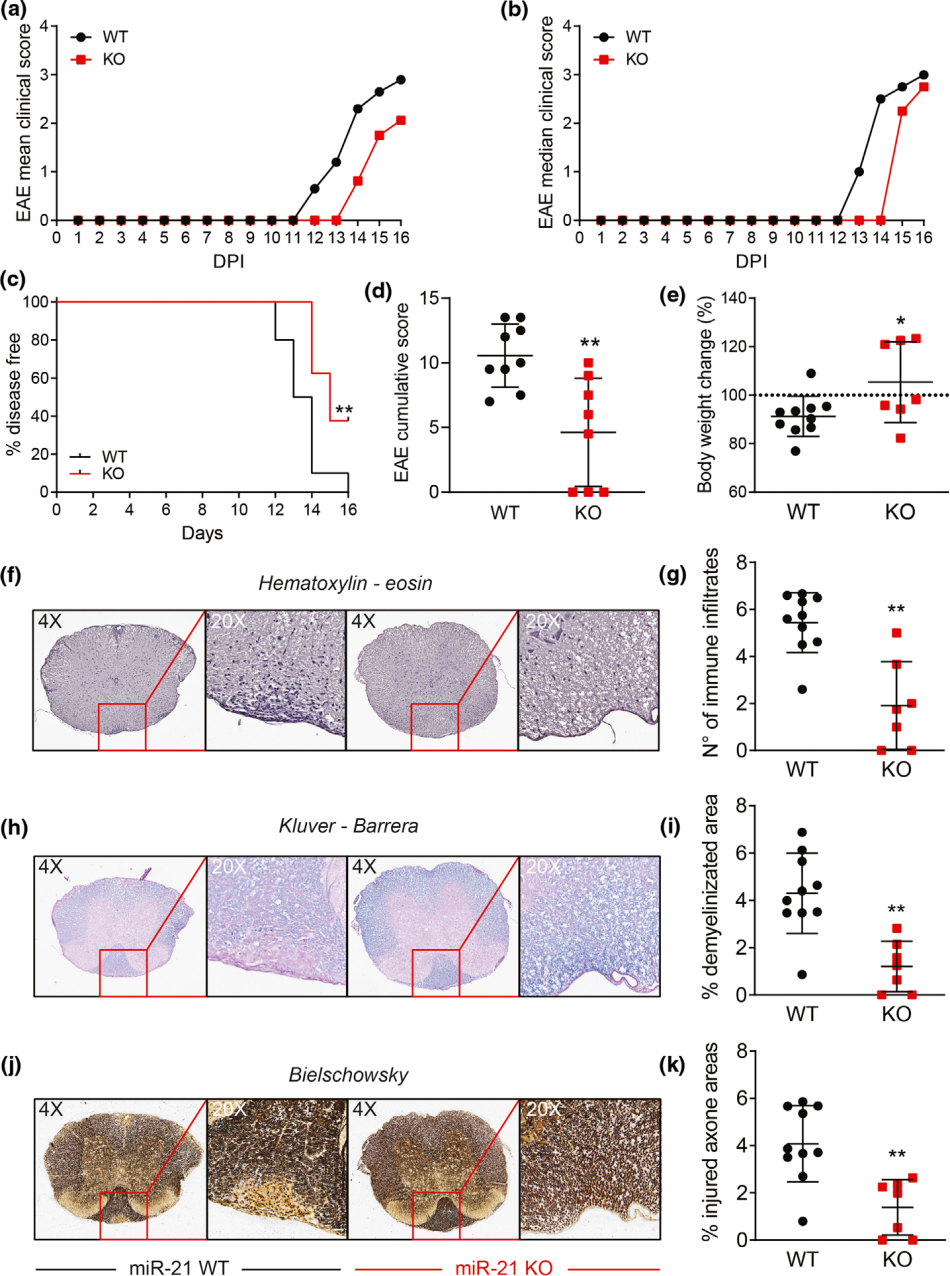
Mice lacking miR‐21 are protected from clinical signs of EAE. WT and KO mice were immunised to induce EAE. Clinical signs of EAE were monitored daily until day 16 post‐immunisation. **(a)** Mean and **(b)** median clinical score and **(c)** disease incidence were assessed for each group. **(d)** EAE was evaluated as cumulative score using Mann–Whitney test (**P ≤ 0.01). **(e)** Percentage of body weight change on day 16 after immunisation. **(f–h)** Neuropathological analysis of **(f–i)** infiltrates, **(g, h)** demyelination and **(j, k)** axonal loss in the spinal cord. Representative stainings are shown in **f**, **g** and **j**; summarising histograms are shown in **i**, **h** and **k**. Data are representative of 3 experiments with 8 or 9 mice per group per experiment. Data represent mean ± SD, statistical significance was tested by the log‐rank (Mantel–Cox) test **(c)** and by the Mann–Whitney test **(d–h)** (*P ≤ 0.05 and **P ≤ 0.01).

Autoreactivity is often caused by T‐cell responses against low‐affinity self‐epitopes that escape central and peripheral tolerance. Previous studies have shown that the compound deletion of miR‐21 from all the cells of mice affected the induction of EAE,^37^ induced via immunisation with the low‐affinity self‐peptide epitope MOG_35–55_ (Figure 4). However, in these mice miR‐21 regulates also DC activation and cytokine production that, in turn, regulates T‐cell response.^38^ To assess the contribution of T‐cell autonomous miR‐21 expression to set the activation threshold for autoimmunity, we compared the induction of EAE in miR‐21 KO vs WT littermates.^39^ The mean (Figure 6a) and median (Figure 6b) clinical scores and the disease incidence (Figure 6c) showed that miR‐21 KO animals were less prone to develop EAE (Figure 6c and d). miR‐21 KO animals had reduced symptomatology, and three out of eight them did not show any measurable clinical sign and weight loss (Figure 6e), an observation reproducible in all three independently performed experiments. Neuropathological findings confirmed that the number of inflammatory infiltrates (Figure 6f and g), demyelination (Figure 6h and i) and axonal damage (Figure 6j and k) in the spinal cord were significantly decreased (Figure 6f, i and k) in miR‐21 KO mice, compared with the miR‐21‐proficient littermates, suggesting an amelioration of the EAE neuropathology in miR‐21 KO mice.

Thus, the lack of miR‐21 expression in T cells resulted in attenuated autoimmunity upon immunisation with a weak self‐antigen.

### miR‐21 KO mice respond normally to LCMV infection

**Figure 7:**
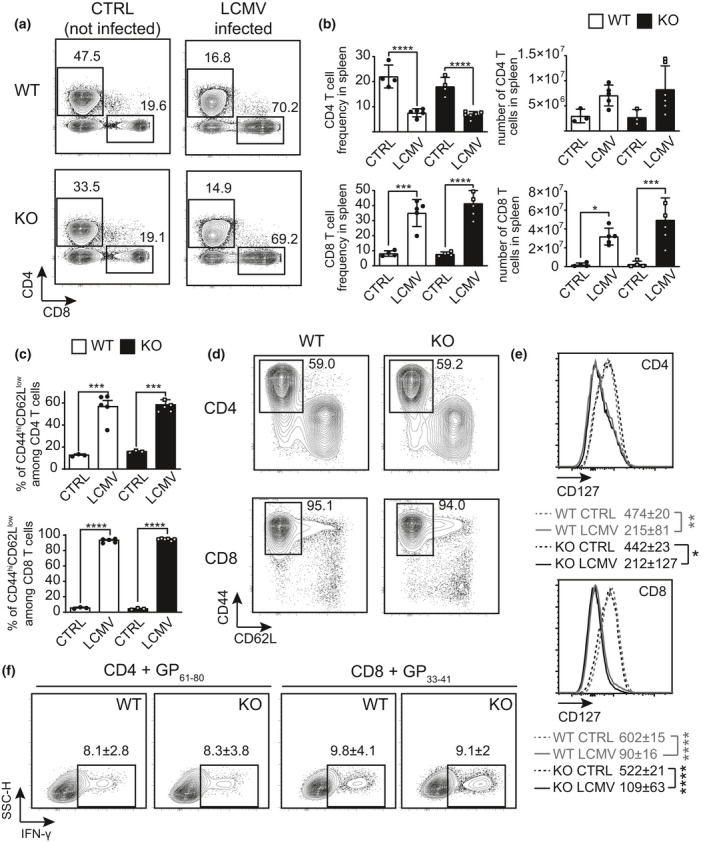
Acute LCMV infection affects equally miR‐21 WT and KO mice. LCMV was injected intravenously at day 0, mice were sacrificed 8 days later, and the spleen was analysed. As a control (CTRL), uninfected mice were sacrificed in parallel. **(a)** CD4/CD8 representative stainings, gated on CD19^−^MHCIa^b−^ cells. **(b)** Summarising graphs of CD4 and CD8 percentages and absolute numbers. **(c)** Frequencies of CD44^hi^CD62L^low^ cells among CD4^+^ or CD8^+^ T lymphocytes. **(d)** Representative stainings of CD44 and CD62L in CD4^+^ or CD8^+^ T cells. **(e)** Histograms depicting CD127 expression in CD4^+^ or CD8^+^ T cells. Numbers represent MFI ± SD. **(f)** IFN‐γ production in CD4^+^ cells restimulated for 5 h with brefeldin A and GP_61–80_ peptide, as well as CD8^+^ cells with GP_33–41_ peptide, both specific to activate LCMV‐restricted T cells. Gates were positioned on empty areas in plots obtained from cells treated with brefeldin A only. One of two comparable experiments, each performed with 5–8 mice per group, is shown. Data in the histograms represent mean ± SD. * *P* ≤ 0.05, **P ≤ 0.01, ***P ≤ 0.001 and ****P ≤ 0.0001 by ANOVA.

The previous results suggested that, unlike the response to low‐affinity antigens, the lack of miR‐21 expression by T cells would have no impact on the T‐cell response against a strong viral epitope, generated in the course of a natural infection. We infected miR‐21‐deficient and miR‐21‐proficient mice with two strains of the LCMV generating the high‐affinity GP_61–80_ peptide epitope (Figure 4), resulting in acute (Armstrong) or persistent (Clone 13) infections, respectively. Upon acute viral infection, 8 days after the administration of Armstrong LCMV, splenic CD8^+^ T cells greatly and similarly expanded upon infection in both types of mice (Figure 7a and b), paralleled by an increase in total splenocyte numbers, even if not significant (Supplementary table 2). Both CD4^+^ and CD8^+^ T cells similarly acquired a CD44^hi^CD62L^low^ CD127^low^ effector memory phenotype (Figure 7c–e). Moreover, a consistent and comparable fraction of CD4^+^ and CD8^+^ T cells from both miR‐21 WT and KO mice acquired the ability to produce IFN‐γ *in vitro* in response to the two LCMV‐derived immunodominant peptide epitopes GP_61–80_ and GP_33–41_, respectively (Figure 7f). Also, at later time points of the acute infection (day 34), when the virus is cleared and the cellular immune response returns to baseline, no quantitative or qualitative differences of LCMV CD4^+^ and CD8^+^ T‐cell response were detected in miR‐21 KO mice (Supplementary figure 9).

The lack of miR‐21 in T cells did not affect the immune response against a chronic viral infection either. At an early time point (day +8) post‐Clone 13 LCMV, circulating CD8^+^ T cells expanded (Supplementary figure 10a, b), as expected in acute viral infections. Both circulating CD4^+^ and CD8^+^ T cells from miR‐21 KO and WT mice responded similarly against the *in vitro* stimulation with the respective strong LCMV epitopes (Supplementary figure 10c). At later time point after chronic infection (day +36), again with no differences between miR‐21 KO and WT mice, the splenic CD4/CD8 ratio returned to baseline levels (Supplementary figure 11a, b), with CD8^+^ T cells showing a significant increase in the T_EM_ fraction (Supplementary figure 11c, d), downregulation of CD127 (Supplementary figure 11e) and exhausted CD44^hi^PD1^hi^ phenotype (Supplementary figure 11g, h), while CD4^+^ T cells exhibited a similar trend without reaching a statistical significance (Supplementary figure 11g, h), in line with the fact that the natural response to LCMV infection is highly dependent on CD8 T cells, in contrast to the CD4 T‐cell dependency for the EAE model.

Together, these results confirmed *in vivo* that miR‐21 has no influence on the regulation of high‐affinity T‐cell response specific for strong xenogeneic peptide epitopes, such as the ones generated by infectious pathogens.

## Discussion

This study defines a new role for miR‐21 as a critical rheostat facilitating the T and iNKT cell response towards low‐affinity antigens, which include also self‐antigens, by sustaining CD28‐dependent costimulation. While on the one hand, this function optimises the breadth of immune surveillance, by extending the capacity of the immune system to respond also to weak stimulators, on the other hand, it exposes the organism to the risk of unleashing T‐cell autoreactivity and autoimmunity. Indeed, we find that mice are more resistant to the induction of the autoimmune pathology EAE, elicited upon immunisation with the weak self‐epitope MOG_35–55_, when T cells lack miR‐21 expression compared with the miR‐21‐sufficient condition. By contrast, miR‐21 is completely dispensable for the induction of CD4^+^ and CD8^+^ T‐cell responses against strong xenoantigens, such as the ones generated by infectious pathogens. Because autoimmune diseases are relatively rare, it is tempting to speculate that miR‐21 must provide evolutionary advantages by promoting efficacious immunity at the population level, at the cost of unleashing autoimmunity in a minority of predisposed subjects, in which tolerance to weak self‐antigens fails upon encountering a triggering event. In this respect, miR‐21 resembles other miRNAs that are upregulated upon T‐cell activation for the positive stimulation of the immune response, such as miR‐17‐92 or miR‐155,^40^ although none of them has been functionally linked to costimulation. We also confirm that miR‐21 has a role either in the control T‐cell survival upon activation *in vitro* or in the selective regulation of the acquisition of T_H_17 effector profile. Furthermore, and in line with previous reports, we find that miR‐21 is largely dispensable for iNKT and T‐cell development, the establishment of their peripheral compartments, and their differentiation into the T_H_0, T_H_1, T_H_2 and, for iNKT cells, also the T_H_17 effector phenotypes.^37^, ^41^ Hence, unlike several other miRNAs that differentially regulate T‐ and iNKT cell development and effector differentiation, miR‐21 exerts a similar function on both subsets.

The current preclinical or clinical evidence positively links miR‐21 with the inflammatory response subtending cancer or chronic inflamed states and autoimmune pathologies such as psoriasis, asthma, EAE/multiple sclerosis, systemic lupus erythematosus and rheumatoid arthritis,^42^ associating it with the acquisition of pathogenic T‐cell effector phenotypes such as T_H_17. Yet, a mechanism of autoimmunity related to miR‐21 impinging on the differential regulation of low‐ vs high‐affinity T‐cell responses was never envisaged before. In addition, we also find that miR‐21 sustains the differentiation *in vitro* of naïve CD4 T cells into potentially pathogenic T_H_17 cells, while it restrains their differentiation into iTregs, promoting conditions that could favour and/or sustain chronic inflammation and autoimmunity. Collectively, therefore, our results confirm and substantially extend the published ones.^7^, ^8^, ^9^, ^10^, ^11^, ^12^, ^43^

miR‐21 is more expressed by activation‐experienced T cells than by naïve T cells,^7^ while our *in vitro* kinetic experiments show that the expression of miR‐21 greatly augmented upon iNKT and T‐cell activation *in vitro* by TCR+CD28 stimulation, in agreement with published reports.^6^, ^44^, ^45^ miR‐21 modulates diverse signalling pathways required for T‐cell activation by directly targeting PDCD4,^4^ PTEN,^3^ FOXO3^46^ and SMAD7^37^ transcripts. Among these targets, we focused on PTEN and PDCD4 because they are particularly relevant for the involvement of miR‐21 in sustaining CD28‐dependent costimulation. Our finding that the lack of miR‐21 expression in T cells results in the dysregulated upregulation of PTEN and PDCD4 upon TCR‐CD28 stimulation is entirely consistent with this involvement. T cells require antigen receptor signalling (signal 1) in the context of CD28 costimulation (signal 2) for optimal activation, cytokine production, proliferation and survival.^32^ CD28 costimulation relies in the downstream activation also of the PI3K‐AKT pathway generating the lipid second messenger phosphatidylinositol‐3,4,5‐triphosphate (PIP_3_).^47^ PIP3 generation is limited by the lipid phosphatase PTEN,^48^ which is upregulated upon TCR+CD28 activation, in this way acting as a negative feedback mechanism that increases the activation threshold of T cells by restraining the CD28‐dependent PI3K pathway activation. Cumulative evidence suggests that PTEN upregulation sets an activation threshold such that weak TCR signalling alone cannot activate PI3K to a level necessary for full T‐cell activation, unless CD28 costimulation overcomes the negative regulation by PTEN.^49^, ^50^ Furthermore, PTEN‐deficient T cells have unconstrained PI3K activity that contributes to costimulation‐independent T‐cell activation, and the development of both autoimmune disease and lymphoma,^51^, ^52^ suggesting that the balance between PI3K and PTEN is critical for regulating appropriate T‐cell activation. Our finding that miR‐21 sets the correct level of PTEN expression in response to CD28 costimulation, permitting T‐cell response to weak TCR signals, fits well with this framework and provides new mechanistic link between CD28 costimulation, the response to low‐affinity self‐antigens and the equilibrium between self‐tolerance and autoimmunity. Interestingly, T‐cell costimulation by CD28 engagement can be replaced by the pharmacological treatment with PMA, which is an inducer of miR‐21 expression, further supporting the role for this miRNA in determining the functional effects of costimulation on T‐cell activation.^9^ Remarkably, miR‐21 has been characterised also for its anti‐apoptotic function in cancer cells,^1^, ^2^ and CD28 costimulation supports T‐cell activation also by delivering anti‐apoptotic signals, mediated in large part by the PI3K pathway member Akt. Our results showing a decreased survival of miR‐21 KO T cell upon CD3+CD28 activation *in vitro*, correlating with an unconstrained PTEN upregulation, would be in line with a defective CD28‐dependent anti‐apoptotic effect resulting from a defective Akt activation and increased cell death. In addition, the activity of the PI3K/Akt signalling pathway is also inhibited by PDCD4,^53^, ^54^ which is another validated target of miR‐21 that has pro‐apoptotic functions by activating BAX and caspases 8, 9 and 3,^55^ in addition of modulating cell division and inflammation. Accordingly, we find that TCR+CD28 activation of miR‐21 KO T cells *in vitro* results in enhanced expression of PDCD4 transcript, compared with WT T cells, consistent with the increased cell death in T cells lacking miR‐21 expression.

Further supporting the evidence that miR‐21 fine‐tunes T‐cell activation threshold via modulation of the CD28 costimulatory pathway, we also showed an impaired upregulation of the EGR1 in miR‐21 KO T cell upon TCR+CD28 activation *in vitro*. Although not a direct target of miR‐21, EGR1 is a transcriptional regulator implicated in development and growth of many cell types, including thymocytes and mature T cells.^56^, ^57^, ^58^ EGR1 gene expression is induced by PI3K signalling,^59^ and in T cells, in line with this, its expression is rapidly upregulated in response to CD28 signalling alone and further increased by combining TCR+CD28 triggering,^35^ helping in turn to lower the threshold for their activation.^34^ EGR1 is also needed for the positive selection of DP thymocytes, which relies on weak antigenic stimulation, further supporting a direct role in sustaining weak TCR signalling.^58^ Therefore, mechanistically, the miR‐21 support to the CD28 costimulatory effects would likely be exerted also via a strong EGR1 upregulation, ultimately resulting in an improved T‐cell response to weak antigenic stimuli.

Previous studies investigated miR‐21 functions in mice in which the miRNA‐containing gene was deleted from the entire organism (miR‐21^−/−^), showing normal lymphoid and myeloid cell development,^37^, ^41^ but a reduced CD4^+^ and CD8^+^ T‐cell proliferation, cytokine production and overall immunosurveillance.^60^ Of note, these miR‐21^−/−^ mice also harboured DCs that produce more IL‐12, compared with WT animals, leading to increased IFN‐γ and decreased IL‐4 production by activated CD4^+^ T cells,^38^ suggesting possible non‐T‐cell autonomous role for miR‐21 in these compound miR‐21 KO mice. In line with this hypothesis, T‐cell proliferation, cytokine production and an overall immunosurveillance were essentially normal in our mice with a selective deletion of miR‐21 in T cells. Thus, the results we have obtained with our miR‐21 KO mice avoid confounding effects and provide a precise information of miR‐21 functions in T cells.

iNKT cells or Tregs constitutively express elevated levels of miR‐21, consistent with their activation‐experienced phenotype, yet, unexpectedly, they are unaffected by the lack of miR‐21 expression. This may be explained by the fact that both these two T‐cell subsets undergo strong agonist selection in the thymus, which should be independent of miR‐21 effects, according to our new results shown here. Furthermore, it is possible that redundant miRNAs may regulate the same transcript targets that are critical during iNKT and Treg cell development. For instance, it has been shown that also miR‐214 targets PTEN transcripts, and this miRNA is upregulated in T cells upon TCR+CD28 stimulation,^61^ suggesting the presence of a potential redundant mechanism of control of PTEN function that would explain the normal T‐ and iNKT cell development and their attenuated, and not completely impaired, functions in the absence of miR‐21 expression.

miR‐21 is expressed in human T cells.^12^ The mature miR‐21 is perfectly conserved in mammals; most importantly, its targeting of PTEN and PDCD4 has been experimentally validated both in human^3^, ^4^ and in murine cells.^62^, ^63^ Collectively, these findings strongly suggest the possibility of the existence of the same post‐transcriptional regulation of low‐affinity T‐cell responses also in human T cells, opening new paths for further research with clinical implications.

In conclusion, our results describe a new T‐cell‐intrinsic role for miR‐21 in facilitating T‐cell responses against poorly immunogenic antigens by sustaining CD28 costimulation while, at the same time, decreasing the threshold for self/non‐self‐discrimination, ultimately impinging on the induction of autoreactive immune responses.

## Methods

### Mice

C57BL/6(N) mice were purchased from Charles River. miR‐21^fl/fl^ mice^26^ were bred with pCD4‐Cre.^64^ iVa14 Tg mice^65^ were purchased from Jackson. All mice were housed in specific pathogen‐free conditions, in roomy cages, allowing free access to food and water. Animal suffering and number were minimised in compliance with the European Communities Council Directive of 24 November 1986 (86/609/EEC). All procedures involving animals were approved by the Institutional Animal Care and Use Committee of the San Raffaele Scientific Institute, Italy (IACUC n° 678).

### Cell staining and flow cytometry

T cells from thymus, spleen and liver were purified and stained as described^16^ using mAbs specific for the following Ags: ROR‐γt, PLZF, FoxP3 (eBioscience, San Diego), HSA/CD24, CD4, CD8, CD62L, NK1.1, TCR‐β, CD44, CD19, MHC‐IA^b^, CD25, IFN‐γ, IL‐4 and IL‐17 (Biolegend, San Diego). Stainings always contained rat anti‐mouse CD16/CD32 Fc blocker 2.4G2 mAb (BD Biosciences, San Jose). iNKT cells were identified by PBS‐57‐loaded mCD1d‐PE tetramers (NIH Tetramer Facility, Atlanta). When needed, cells were counted with Flow‐Count Fluorospheres (Beckman Coulter, Brea). Proliferation was analysed by labelling cells at day 0 with 0.5 μm 5(6)‐carboxyfluorescein diacetate *N*‐succinimidyl ester (CFSE; Sigma‐Aldrich, St. Louis). Apoptosis was detected by fluorescent CaspACE FITC‐VAD‐FMK (Promega, Madison) or with Annexin V (BD Biosciences) and DAPI (Santa Cruz, Santa Cruz). Dead cells and doublets were excluded with DAPI or LIVE/DEAD fixable dead cell (Invitrogen, Carlsbad) staining and physical gating. Intranuclear staining for transcription factors (FoxP3, PLZF, RORγt, all mAbs from eBioscience) was performed using the Foxp3/Transcription Factor Staining Buffer Set (eBioscience). Intracellular staining was performed fixing the cells with 2% paraformaldehyde (Sigma‐Aldrich) and permeabilised with Perm/Wash (BD Biosciences). Samples were acquired on FACSCanto II flow cytometer (BD Biosciences) and data analysed by FlowJo software (Tree Star Inc, Ashland). Gating strategies are depicted in Supplementary figure 12.

### Isolation and purification of cells by sorting

To sort thymic iNKT cells, mature thymocytes were obtained by depleting HSA^+^ cells with B2A2 rat anti‐mouse monoclonal Ab (mAb) plus Low‐Tox‐M Rabbit Complement, followed by a lympholyte M (both from Cedarlane Laboratories, Burlington) gradient.^16^ Resulting cells were stained with anti‐TCRβ plus the indicated mAbs and CD1d tetramers, and sorted (MoFlo; Beckman Coulter) excluding cell debris and doublets. The purity of sorted fractions was checked by flow cytometry reanalysis.

Naïve CD4^+^ T cells were isolated from spleen and cervical/inguinal LN with the Naive CD4^+^ T Cell Isolation Kit, mouse (Miltenyi Biotec, Bergisch Gladbach) according to the manufacturer's instructions.

### T‐ and iNKT cell activation

For pri‐miR‐21 and miR‐21 quantification, total T cells, or naïve CD4^+^ T cells, or iNKT cells were enriched by immunomagnetic sorting from the spleen of C57BL/6N mice and iVa14 Tg mice, respectively, and activated with Dynabeads Mouse T‐Activator CD3/CD28 (Thermo Fisher, Waltham) at 1 cell:1 beads ratio in complete RPMI medium supplemented with 30 U mL^−1^ hrIL‐2 (Chiron, Emeryville) at 37°C for the indicated times. Cells were then harvested, and RNA was extracted. For Ag‐specific iNKT cell activation *in vivo*, 4 μg α‐GalCer (Avanti Polar, Alabaster) was injected i.p. into mice. Sera were collected 2 days before and 2 and 6 h after α‐GalCer injection, and IL‐4 and IFN‐γ production was tested by ELISA. Otherwise, mice were sacrificed at 2 h after the α‐GalCer injection and IL‐4 and IFN‐γ production in splenic iNKT cells was evaluated by intracellular staining.

For Ag‐specific iNKT follicular helper cell induction *in vivo*, C57BL/6N mice were immunised s.c. in the left flank at day 0 with and and Ag mix dissolved in PBS or mixed with either 100 ng α‐GalCer or Imject Alum Adjuvant (Thermo Fisher). The Ag mix consisted for each dose of 50 μg 4‐hydroxy‐3‐nitrophenyl‐chicken γ globulin (NP‐CGG; Biosearch Technologies, Hoddesdon), 50 μg ovalbumin (OVA, Sigma‐Aldrich), 3 μg tetanus toxoid (Novartis Vaccine, Basel), 3 μg bovine serum albumin fraction V (BSA, Roche, Basel) and 3 μg keyhole limpet haemocyanin (KLH; endotoxin‐free; Calbiochem, San Diego). Mice were sacrificed at day +7, and iNKT_FH_ cell differentiation was evaluated on spleen and LN cells as described.^27^

For Ag‐specific iNKT cell activation *in vitro*, the frequency of splenic iNKT cells was assessed by flow cytometry for each mouse and the corresponding numbers of splenocytes containing 5000 iNKT cells were seeded in each well of 96‐well plates. Cells were activated in RPMI medium + 10% FCS containing twofold increasing concentrations (from 3 to 800 ng) of α‐GalCer and OCH (NIH Tetramer Facility). After 24 h, supernatants were collected and cytokine production was assessed by ELISA.

For Ag non‐specific activation of iNKT or T cells *in vitro*, 2 × 10^6^ total spleen or LN cells or hepatic mononucleated cells were cultured for 4 h in the presence of 25 ng mL^−1^ PMA + 1 µg mL^−1^ ionomycin (Sigma‐Aldrich), plus 10 μg mL^−1^ brefeldin A (Golgi Stop; Sigma‐Aldrich) for the last 2 h. Not activated controls only had brefeldin A for the last 2 h of culture. Cytokine production was detected by cytofluorimetric analysis.

For Ag non‐specific T helper cell polarisation *in vitro*, 5 × 10^5^ purified naïve CD4^+^ T cells were cultured for 4 days with plate‐bound 2 μg mL^−1^ anti‐CD3 + 2 μg mL^−1^ anti‐CD28 mAbs (145‐2C11 and 37.51 mAbs, UltraLEAF, Biolegend), plus the following supplements: nil for T_H_0; 10 ng mL^−1^ mrIL‐12 + 50 U mL^−1^ hrIL‐2 + 1 μg mL^−1^ anti‐IL‐4 mAb for T_H_1*;* 10 ng mL^−1^ mrIL‐4 + 50 U mL^−1^ hrIL‐2 + 1 μg mL^−1^ anti‐IFN‐γ for T_H_2; and 25 ng mL^−1^ mrIL‐6 + 2 ng mL^−1^ TGF‐β1 + 20 ng mL^−1^ rmIL‐1β + 20 ng mL^−1^ mrIL‐23 for T_H_17 (cytokines; R&D Systems, Minneapolis; and blocking mAbs; BioXcell, Lebanon). At day 4, cells were harvested and restimulated for 4 h in the presence of 25 ng mL^−1^ PMA + 1 µg mL^−1^ ionomycin (Sigma‐Aldrich), plus 10 μg mL^−1^ brefeldin A (Golgi Stop; Sigma‐Aldrich) for the last 2h and analysed by intracellular staining as described above.

For iTreg differentiation, 5 × 10^5^ purified naïve CD4^+^ T cells were cultured for 4 days with plate‐bound 10 μg mL^−1^ anti‐CD3 + soluble 5 μg mL^−1^ anti‐CD28 mAbs + 50 U mL^−1^ rhIL‐2 + 5 ng mL^−1^ rmTGF‐β. At day 4, cells were harvested and stained and analysed by intranuclear staining as described above.

For Ag non‐specific CD4 T‐cell activation *in vitro*, purified CD4^+^ T cells were plated (5 × 10^4^ cells/well in RPMI medium + 10% FCS) in 96‐well plates precoated with serial 1:2 dilutions of anti‐CD3 145‐2C11 mAb ranging from 6400 to 3.1 ng, with or without 2 μg mL^−1^ anti‐CD28 37.51 mAb, or vice versa with of anti‐CD28 mAb ranging from 6400 to 3.1 ng, with or without 2 μg mL^−1^ anti‐CD3 mAb (both Biolegend). After 24‐h activation, supernatants were collected and cytokine production was assessed by ELISA.

### ELISA

ELISA plates (Nunc) were coated with of rat anti‐mouse IFN‐γ AN18, rat anti‐mouse IL‐4 11B11 or rat anti‐mouse IL‐2 JES6‐1A12 mAb (BD Biosciences). After blocking, samples were added for 2 h followed, after extensive washing, by biotin‐labelled R4‐6A2, BVD6‐24G2 or JES6‐5H4 mAb specific for each cytokine. Signal was revealed by streptavidin‐bound horseradish peroxidase and TMB substrate (Sigma‐Aldrich). Absorbance was read at 450 nm optical density, and cytokine concentration was calculated based on the standard curve.

### Measurement of Ag‐specific Ab titres

For Ag‐specific Ab titration, following collection of preimmunisation sera, miR‐21 KO mice and negative littermate mice were immunised at day 0 by a s.c. injection of 100 μg of OVA (Sigma‐Aldrich) mixed with 100 ng of α‐GalCer (Avanti Polar). Blood was drawn by retro‐orbital phlebotomy after +7, +14 and +23 days to determine specific Ig titres of the primary responses on sera. On day +23, all mice were boosted with 100 μg OVA dissolved in PBS, and the secondary Ig responses were determined at days +36 and +49. Individual sera were titrated in parallel at the same time for their Ag‐specific Ig content by end‐point ELISA as described.^27^ Antibody titres were expressed as reciprocal dilutions giving an OD450 > mean blank OD450 + 3 SD. Blanks consistently displayed OD450 < 0.1 and < 10% variability.

### RNA extraction and quantitative real‐time RT‐PCR

Total RNA from sorted cells was isolated with the mirVana Kit (Thermo Fisher) and quantified by NanoDrop spectrophotometer (Thermo Fisher). RNA quality and integrity were verified by an Agilent 2100 Bioanalyzer profile using Agilent RNA 6000 Pico or Nano kits.

miR‐21‐specific reverse transcription was performed using TaqMan MicroRNA Reverse Transcription Kit (Thermo Fisher). qRT‐PCR was performed using TaqMan MicroRNA Assay Mix containing PCR primers and TaqMan probes (Thermo Fisher). Expression values were normalised to snoRNA‐202. For pri‐miR‐21 expression, RNA samples were treated with Turbo DNA‐FREE Kit (Thermo Fisher) to get rid of any possible contaminant DNA. Retrotranscription was then performed with the High Capacity cDNA Reverse Transcription Kit (Thermo Fisher). qRT‐PCR was performed using TaqMan Pri‐miRNA Assay mix containing PCR primers and TaqMan probes specific for pri‐miR‐21 (Applied Biosystems) or TaqMan Gene Expression assays (Thermo Fisher; Egr1, Mm00656724_m1; PTEN, Mm00477208_m1; and PDCD4, Mm01266062_m1). Expression values were normalised to GAPDH (Mm99999915_g1) for pri‐miR‐21, and to HPRT (Mm03024075_m1) for mRNAs.

qRT‐PCR was performed in triplicate with 10–50 ng cDNA/reaction using TaqMan Universal PCR Master Mix, no AmpErase UNG (Thermo Fisher) on ABI Prism 7900 cycler (95°C for 15 s and 60°C for 1 min, 40 cycles), and analysed with SDS 2.2.1 software. The relative quantification of gene expression was determined by the comparative Ct method.

### Immunisation with synthetic peptides and induction of EAE

To assess T‐cell response to low‐ and high‐affinity peptide epitopes, separate groups of C57BL/6N or miR‐21 KO mice were immunised s.c. with either 200 μg of MOG_35–55_ (Espikem, Prato) or 200 μg of GP_61–80_ (ProteoGenix, Costa Mesa) emulsified in Complete Freund's Adjuvant (Sigma‐Aldrich) supplemented with 4 mg mL^−1^ heat‐killed Mycobacterium tuberculosis (strain H37Ra; Difco, Franklin Lakes). After 10 days, splenocytes were collected and restimulated (2 × 10^6^ cells per well) in 96‐well plates for 5 h with twofold dilutions (from 10 to 0.625 μm) of the respective cognate peptide in the presence of brefeldin A, stained intracellularly for IFN‐γ production and analysed by flow cytometry.

EAE induction was obtained by adding two 500 ng pertussis toxin through i.v. injections on days 1 and 3 according to the protocol described above for MOG. EAE was evaluated clinically daily and histologically (with haematoxylin and eosin to detect inflammatory infiltrates; Kluver–Barrera to detect demyelination and Bielschowsky to detect axonal damage) as previously described.^39^

### Viral infections

LCMV was propagated and quantified as described^66^ and dissolved in 200 μL of PBS prior to intravenous injection. A single dose of 1 × 10^6^ PFU was used to induce infection. Cells analysed for cytokine production, prior to intracellular staining, were stimulated for 5 h at 37°C with brefeldin A alone as a negative control or with brefeldin A + either 2 μg mL^−1^ GP_61–80_ for CD4 T cells or 2 μg mL^−1^ GP_33–41_ for CD8 T cells (ProteoGenix).

### Statistical analysis

All statistical tests were conducted with GraphPad Prism. Comparisons between two groups were done with the two‐tailed Student *t*‐test for unpaired samples, applying correction for unequal variances when required, except for EAE where non‐parametric Mann–Whitney *U*‐tests were used. The Kaplan–Meier survival curves and log‐rank test were used to compare day of onset. Comparisons between three or more groups were performed with one‐way ANOVA plus the Tukey post‐test. For all tests, a value of *P* < 0.05 was considered significant.

## Conflict of interest

The authors declare no conflict of interest.

## Author contributions

**Maya Fedeli:** Conceptualization; Data curation; Formal analysis; Funding acquisition; Investigation; Project administration; Writing‐original draft; Writing‐review & editing. **Mirela Kuka:** Investigation; Methodology. **Annamaria Finardi:** Investigation; Methodology. **Francesca Albano:** Investigation. **Valentina Viganò:** Investigation. **Matteo Iannacone:** Methodology; Resources. **Roberto Furlan:** Methodology; Resources. **Paolo Dellabona:** Conceptualization; Funding acquisition; Resources; Supervision; Writing‐original draft; Writing‐review & editing. **Giulia Casorati:** Conceptualization; Funding acquisition; Resources; Supervision; Writing‐original draft; Writing‐review & editing.
