# Supplementary material for: miR‐21 sustains CD28 signalling and low‐affinity T‐cell responses at the expense of self‐tolerance
Source: Clin Transl Immunology. 2021 Sep 21;10(9):e1321. doi: 10.1002/cti2.1321 (PMC8454917; doi:10.1002/cti2.1321)
Supplement: Supplementary file 1 [file CTI2-10-e1321-s001.pdf]

Supporting information

Supplementary figures

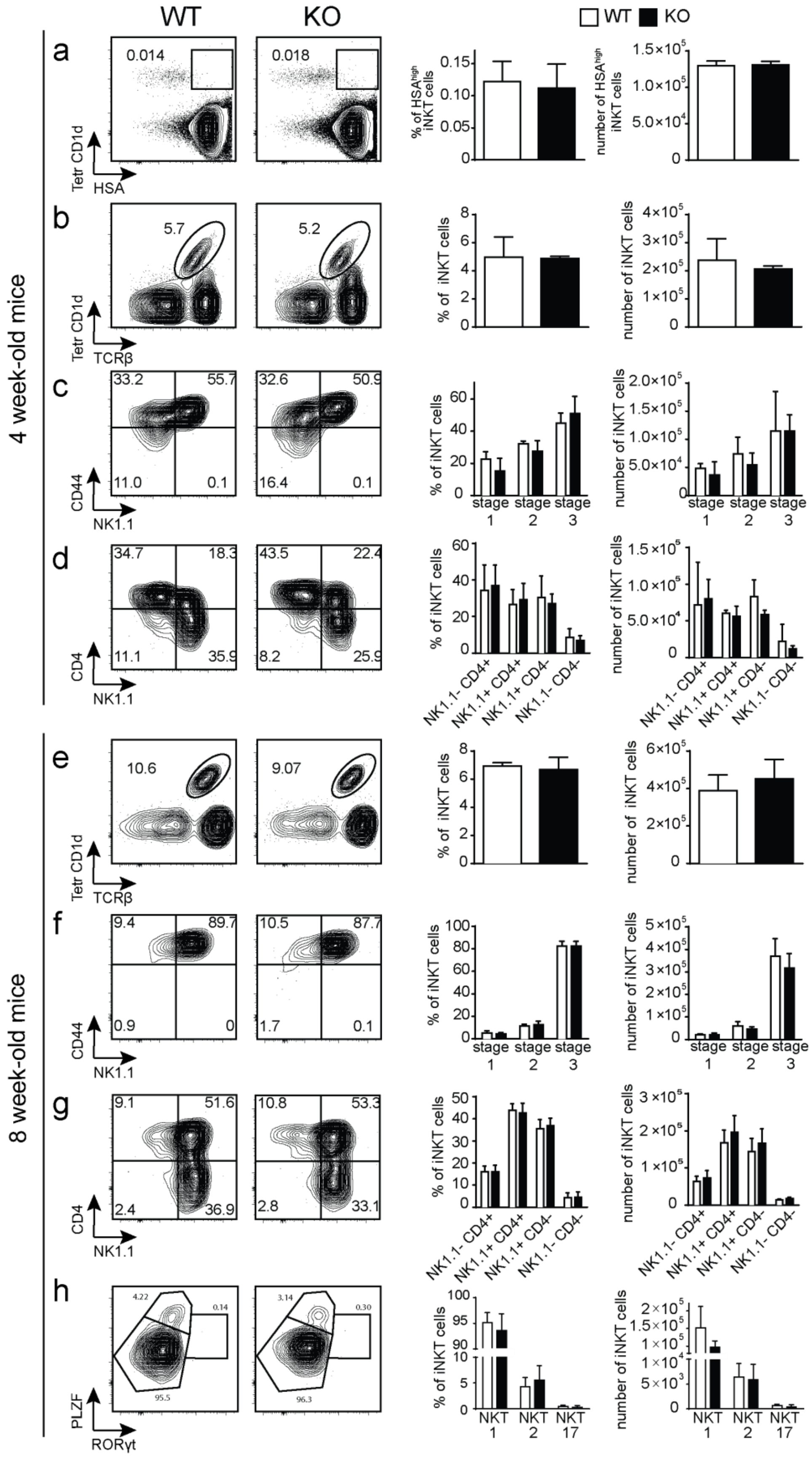

Supplementary figure 1

Supplementary figure 1. iNKT cell development is correct in miR-21 KO mice. Thymic cells were obtained from miR-21 WT and KO mice at 4 **(a–d)** and 8 weeks of age **(e–h)**; representative plots are shown on the left and summarizing graphs with frequencies and cell numbers are on the right. **(a)** Stage 0 iNKT cells were identified as HSA^{high}Tetr $CD1d^{+}$ among viable thymocytes. (**b**, **e)** Plots are gated on HSA^{low} cells, iNKT cells are identified as $TetrCD1d^{+}TCR\text{-}\beta^{+}$. **(c, d)** and **(f, g)** Plots are gated on iNKT cells, showing iNKT cell subdivision in stages (**c**, **f)** and subsets **(d**, **g)**, by the expression or NK1.1 and CD44 or NK1.1 and CD4, respectively. **(h)** Subdivision into NKT1 ($PLZF^{low}ROR\gamma t^{-}$), NKT2 ($PLZF^{high}ROR\gamma t^{-}$), and NKT17 ($PLZF^{int}ROR\gamma t^{+}$); plots are gated on iNKT cells. Data representative of 2 or 3 experiments with 3 or 4 mice per experiment. Histograms data represent mean ± SD, statistical significance was tested by a two-tailed unpaired *t*-test in **(a)**, **(b)**, and **(e)**, by ANOVA in **(c)**, **(d)**, **(f)**, **(g)** and **(h**).

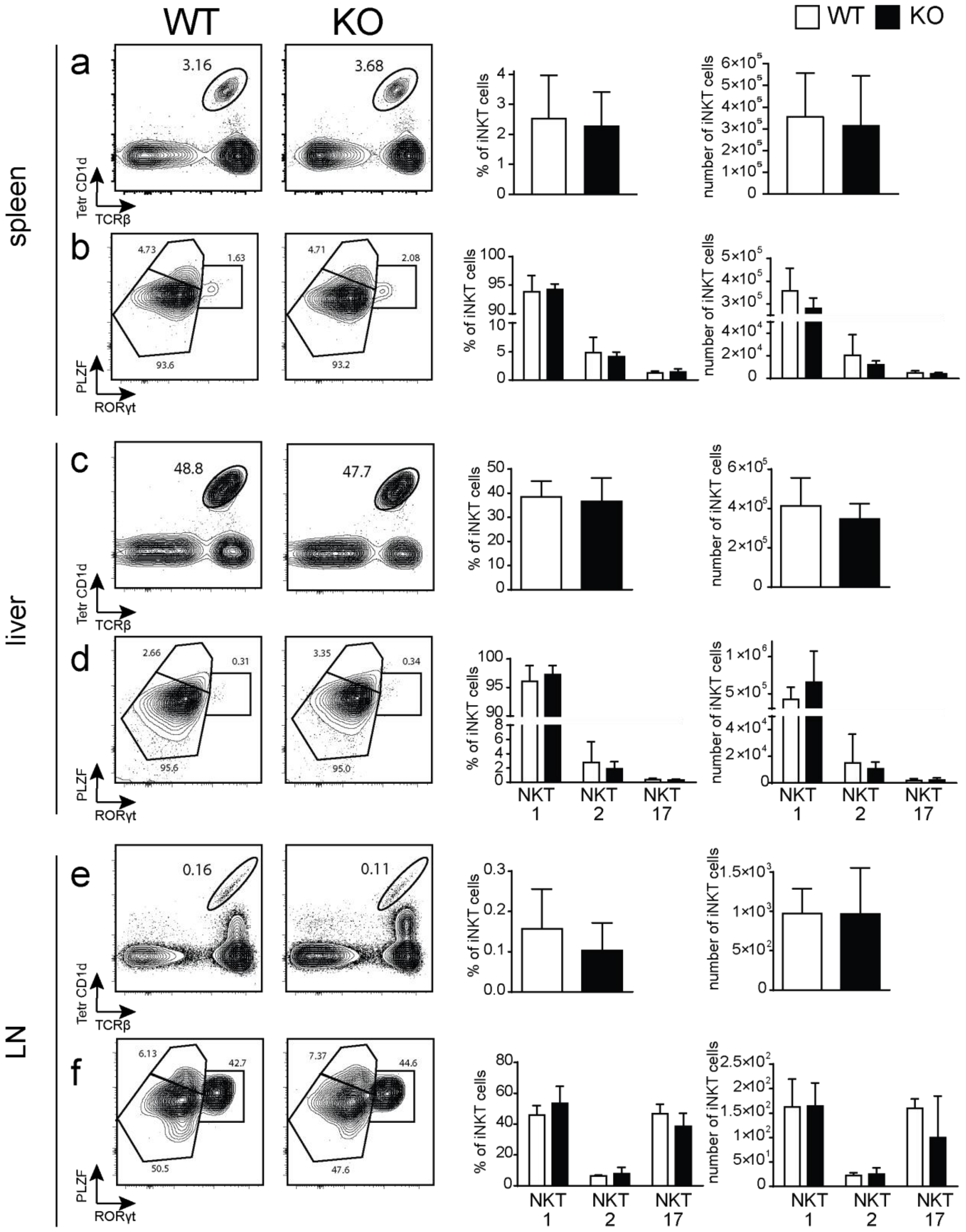

Supplementary figure 2

Supplementary figure 2. iNKT cell distribution in functional subsets and peripheral organs is maintained. Peripheral lymphocytes of miR-21 KO mice, purified from spleen **(a, b)**, liver **(c, d)** and a pool of inguinal and cervical LN **(e, f)**; representative plots are shown on the left and summarizing graphs with frequencies and cell numbers are on the right. **(a**, **c**, **e)** Plots are gated on $CD19^{-}$ $MHC\text{-}IA^{b-}$ cells, iNKT cells are identified as $TetrCD1d^{+}TCR\text{-}\beta^{+}$. **(b**, **d**, **f)** Subdivision into NKT1 ($PLZF^{low}ROR\gamma t^{-}$), NKT2 ($PLZF^{high}ROR\gamma t^{-}$), and NKT17 ($PLZF^{int}ROR\gamma t^{+}$); plots are gated on iNKT cells. Data representative of 2 or 3 experiments with 3 or 4 mice per experiment. Histograms data represent mean ± SD, statistical significance was tested by a two-tailed unpaired *t*-test in **a**, **c** and **e**, by ANOVA in **b**, **d** and **f**.

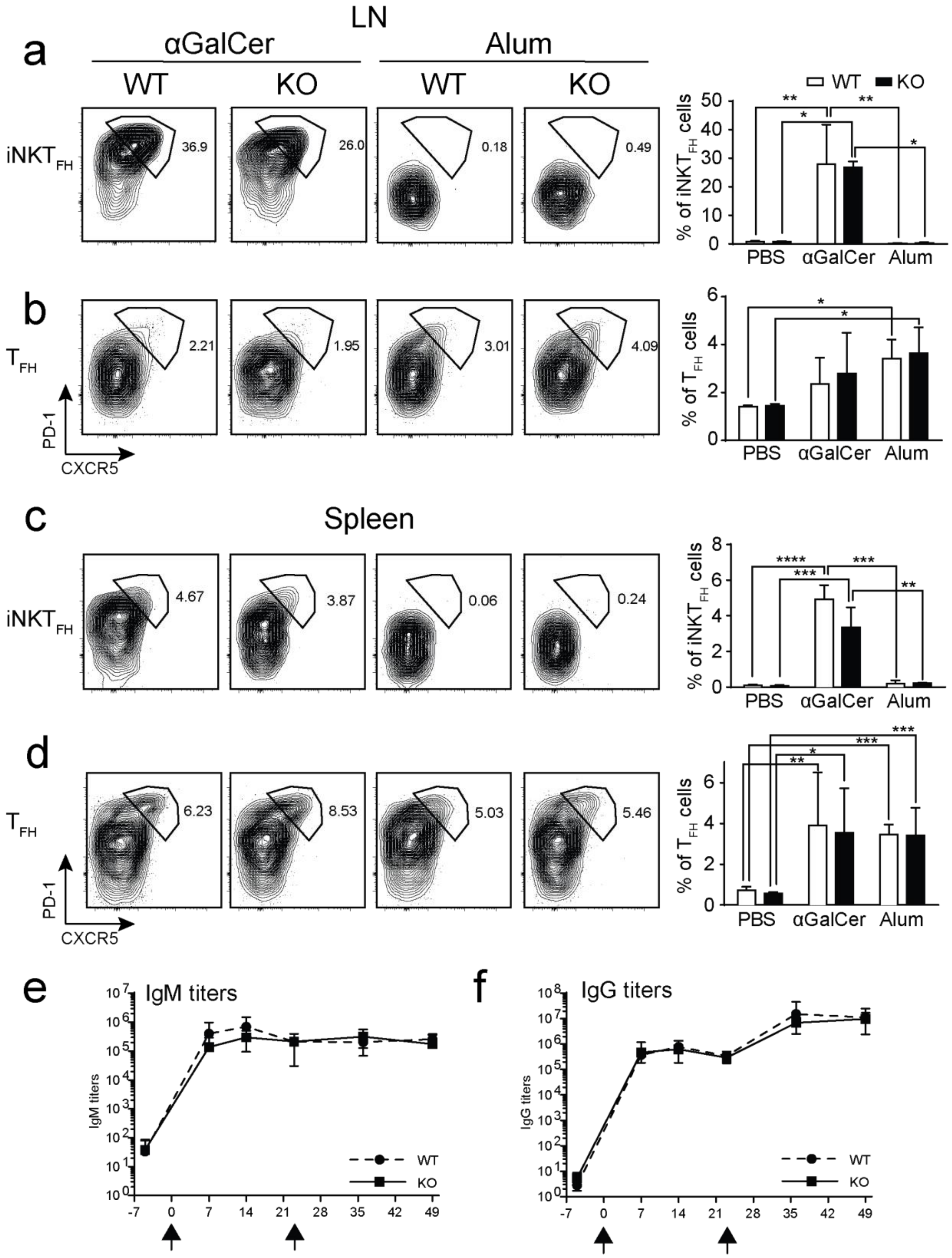

Supplementary figure 3

Supplementary figure 3. Immunization induces a normal iNKT$_{FH}$ and T$_{FH}$ cell differentiation in miR-21 KO mice. (a–d) Spleen **(a, b)** and LN **(c, d)** cells from miR-21 WT and KO mice obtained 7 days after immunization with α-GalCer or Alum and a mixture containing OVA, NP-CGG, BSA, KLH and Tetanus toxoid. Cells were stained to assess CXCR5 and PD-1 upregulation among TetrCD1d^{+}TCR-β^{+} iNKT cells **(a**, **c)** or TCR-β^{+}CD4^{+}CD44^{high} Ag-experienced T cells **(b**, **d)**. Representative plots are shown on the left, gates were positioned on empty areas in plots obtained from mice injected with Ag + PBS. Summarizing histograms on the right represent mean ± SD. * $P \leq 0.05$ ** $P \leq 0.01$, *** $P \leq 0.001$, **** $P \leq 0.0001$ by ANOVA test. One of 3 comparable experiments, each performed with 3 or 4 mice/group, is shown. **(e, f)** Ag-specific Ab titers of sera collected from mice immunized (when indicated by the arrows) first with OVA + α-GalCer as an adjuvant and second with OVA + PBS. IgM **(e)** and IgG **(f)** titers were measured. Data are representative of 2 experiments with 5-7 mice per experiment.

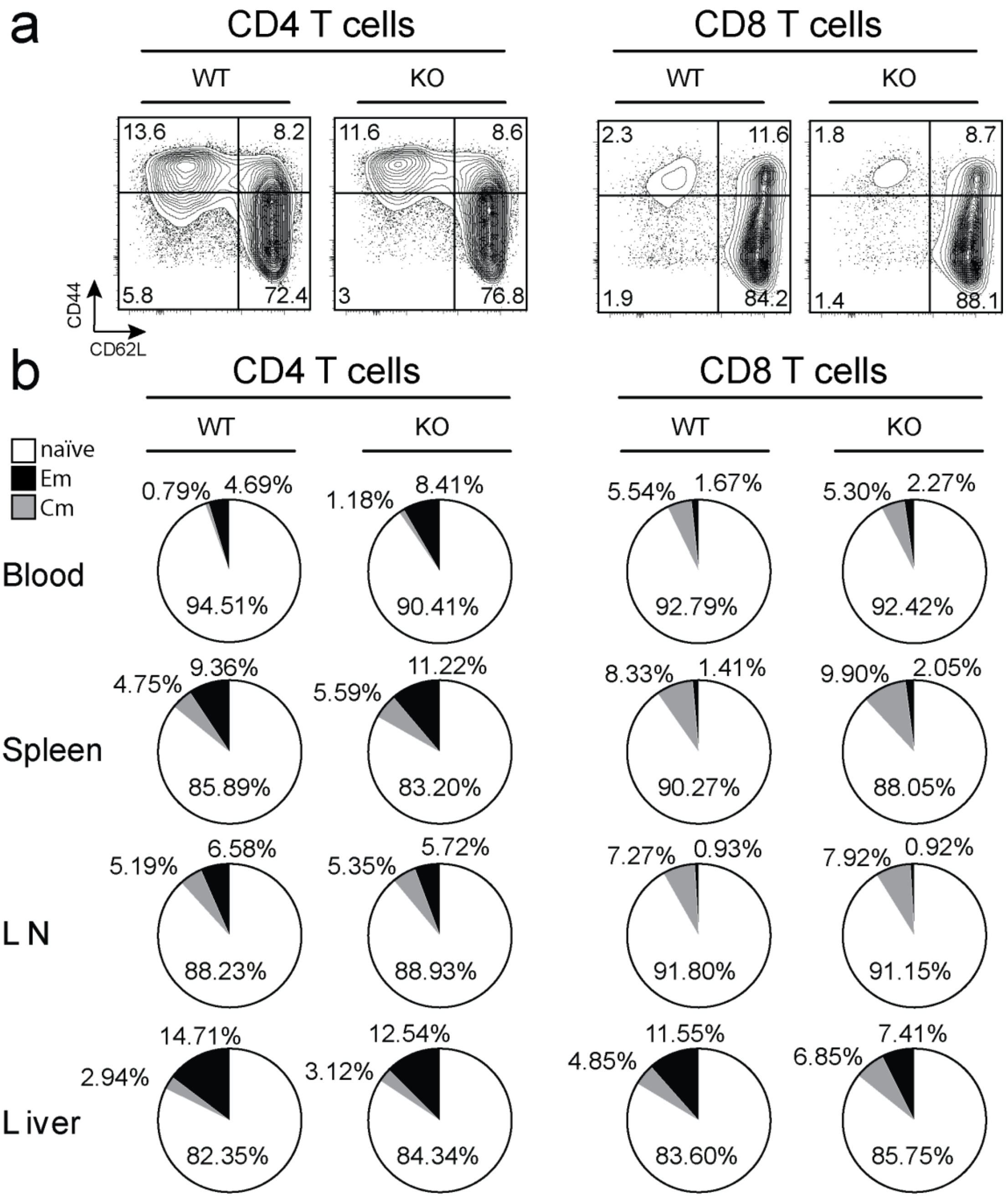

Supplementary figure 4. $T_{Naïve}$, T_{EM}, and T_{CM} phenotype in miR-21 KO mice does not show any alteration. (a) representative plot of splenic CD4$^+$ (gated as or TCR-β$^+$CD4$^+$) or CD8$^+$ (gated as or TCR-β$^+$CD8$^+$) T cells. $T_{Naïve}$, (CD44^{low}, CD62L^{high}), T_{CM} (CD44^{high}, CD62L^{high}), and T_{EM} (CD44^{high}, CD62L^{low}) were identified. **(b)** Pie charts of $T_{Naïve}$, T_{EM}, and T_{CM} CD4 and CD8 cells, in blood, spleen, LN and liver of miR-21 WT and KO mice. Data representative of 3 experiments with 3 or 4 mice per experiment. Statistical significance was tested by ANOVA.

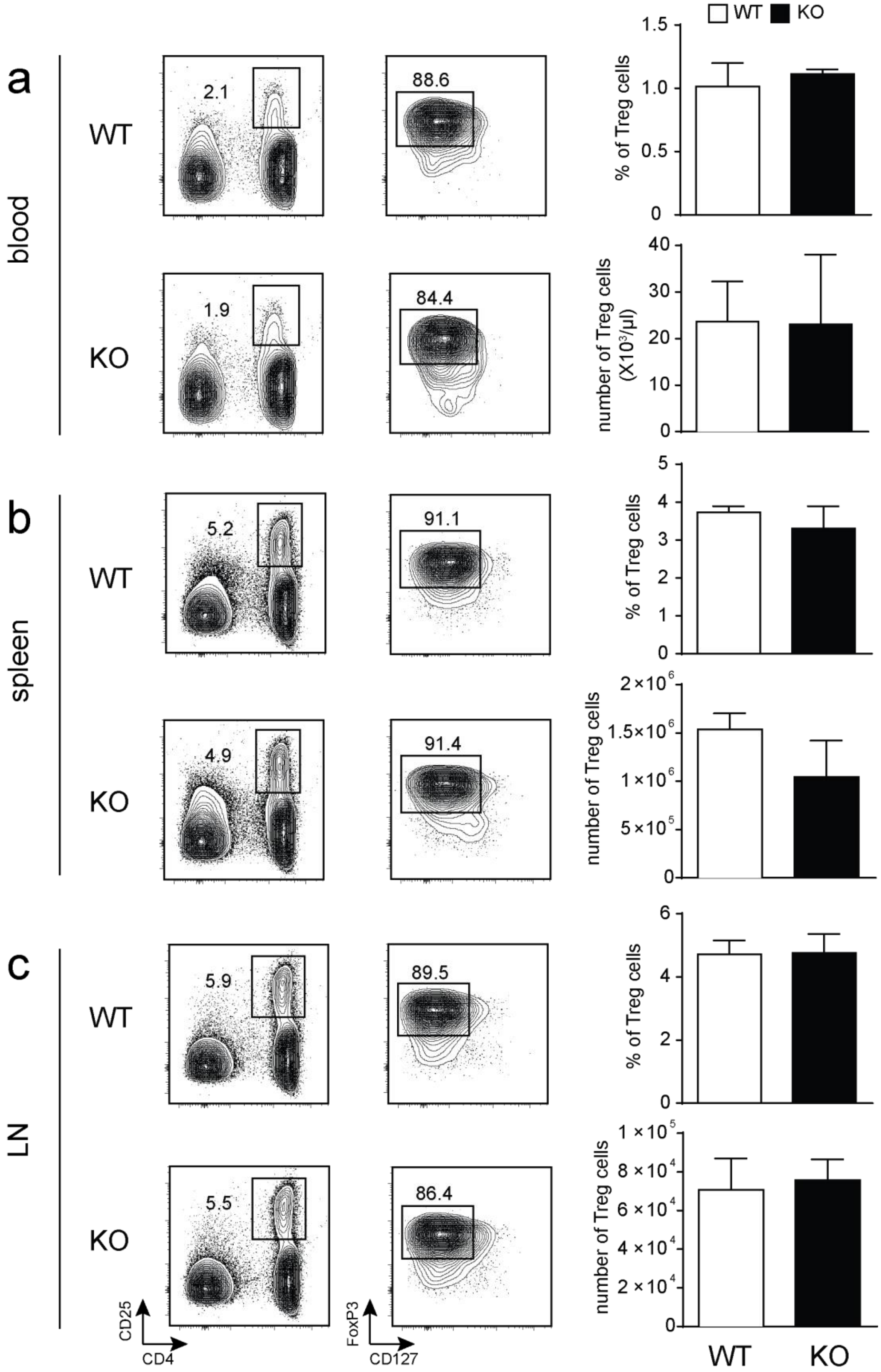

Supplementary figure 5

Supplementary figure 5. Tregs develop correctly in miR-21 KO mice. Tregs were identified as FoxP3$^+$CD127$^-$ cells, gated on CD4$^+$CD25$^+$cells identified among TCR-β$^+$ lymphocytes. Representative plots are shown on the left, summarizing graphs of percentages and absolute numbers of Tregs are on the right. Tregs were analyzed in **(a)** blood, **(b)** spleen, and **(c)** LN. Histograms data represent mean ± SD. Data representative of 3 experiments with 3 or 4 mice per experiment. Statistical significance was tested by a two-tailed unpaired *t*-test.

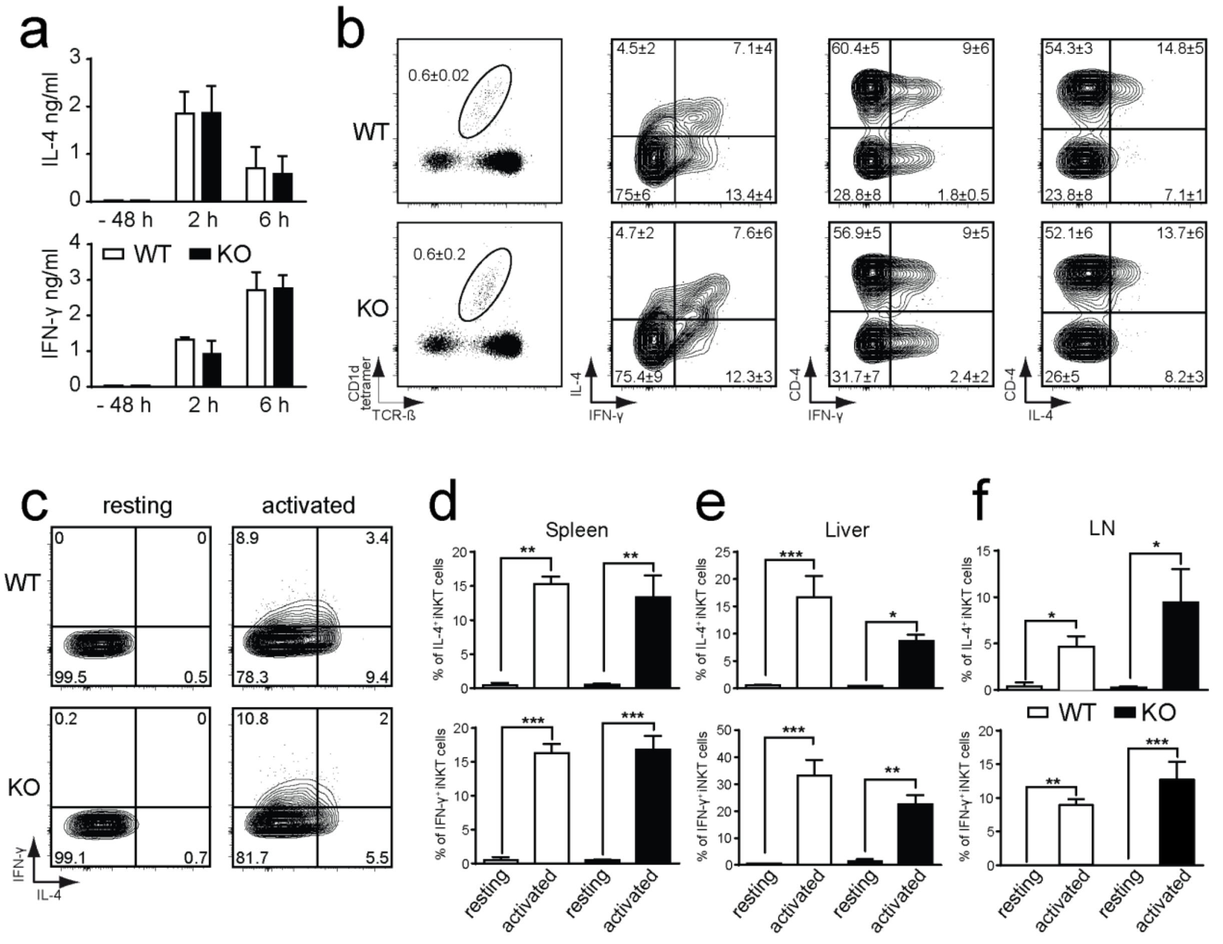

Supplementary figure 6. miR-21 KO iNKT cells respond correctly to cell activation. (a, b) *In vivo* activation of iNKT cells of miR-21 KO and WT mice. Mice were injected i.p. with 4 µg of α-GalCer. **(a)** Sera were collected 2 days before the injection, 2 and 6 hours after. IL-4 and IFN-γ production was tested by ELISA. Histograms data represent mean ± SD**. (b)** Mice were sacrificed 2 h after the injection. IL-4 and IFN-γ production was evaluated by intracellular staining of splenic iNKT cells. iNKT cells are identified as TetrCD1d^{+}TCR-β^{+} among the CD19^{-} MHC-IA^{b-} population. Cytokine stainings are gated on iNKT cell. Means ± SD are shown. **(c–f)** *In vitro* activation for 4 h with PMA/ionomycin of cells purified ex-vivo from spleen, liver and LN of miR-21 KO and WT mice. Cytokine production was evaluated by intracellular staining, in iNKT cells selected through gating. **(c)** Representative plots of splenic resting and activated iNKT cells. Summarizing graphs of cytokine production by **(d)** splenic, **(e)** hepatic and **(f)** lymph nodal iNKT cells. Histograms data represent mean ± SD. Data are representative of 2 experiments with 3 or 4 mice per experiment. * $P \leq 0.05$ ** $P \leq 0.01$, *** $P \leq 0.001$ by ANOVA.

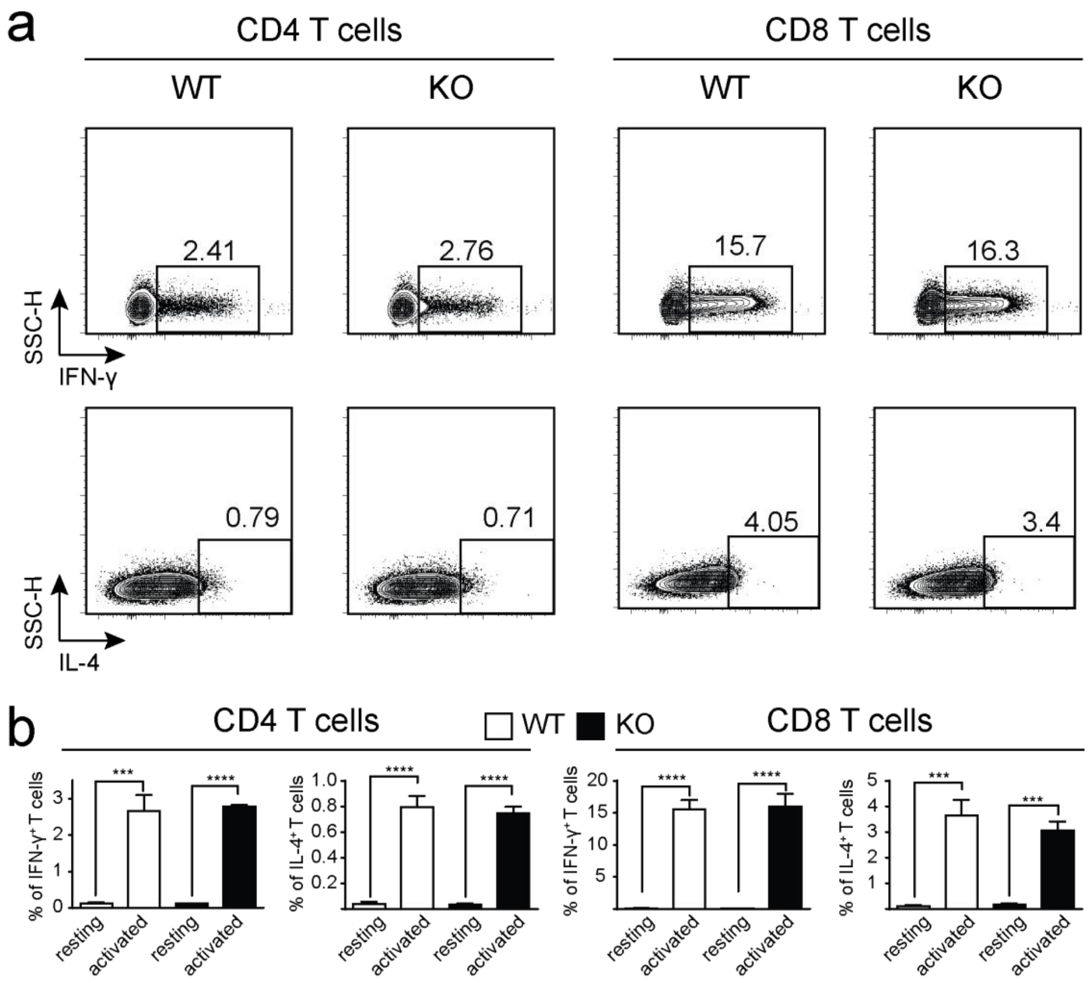

Supplementary figure 7. miR-21 KO T cells produce normal amount of cytokines upon activation. *In vitro* activation for 4 h with PMA/ionomycin of splenic cells purified ex-vivo. IL-4 and IFN-γ production was evaluated by intracellular staining. **(a)** Representative plots of activated $CD4^+$ (gated as or TCR-β^+CD4^+) or $CD8^+$ (gated as or TCR-β^+CD8^+) T cells. Gates were placed accordingly to result empty in not activated (resting) samples. **(b)** Summarizing graphs of IL-4 and IFN-γ production by splenic CD4 and CD8 T cells. Histograms data represent mean ± SD. Data are representative of 2 experiments with 4 mice per experiment. Statistical significance was tested by ANOVA.

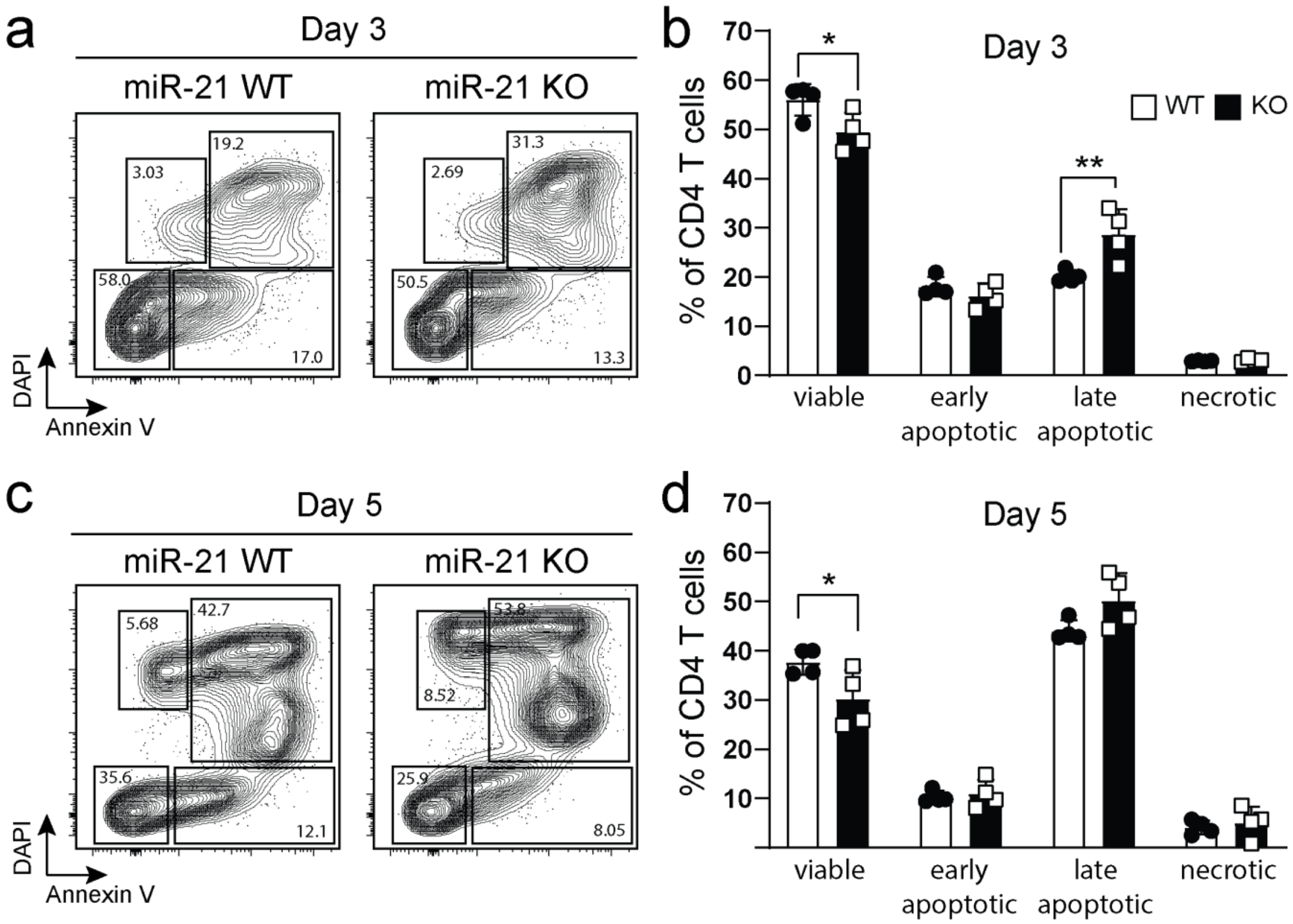

Supplementary figure 8. miR-21 KO CD4 T cells die early of apoptosis. Naïve CD4 T cells, purified from miR-21 WT and KO spleens and LN, were cultured *in vitro* for 3 or 5 days stimulated with anti-CD3 and anti-CD28 only. Cells in culture were stained with Annexin V and DAPI identifying viable cells as Annexin V^- $DAPI^-$, early apoptotic cells as Annexin V^+ $DAPI^-$, late apoptotic cells as Annexin V^+ $DAPI^+$, and necrotic cells as Annexin V^- $DAPI^+$. **(a, b)** Representative plots and summarizing graphs after 3 days in culture. **(c, d)** representative plots and summarizing graphs after 5 days in culture. Data are representative of 2 experiments with 4 mice per experiment. Histograms data represent mean ± SD, statistical significance was tested by two-way ANOVA in **(b)** and **(d)** (*$P \leq 0.05$ ** $P \leq 0.01$).

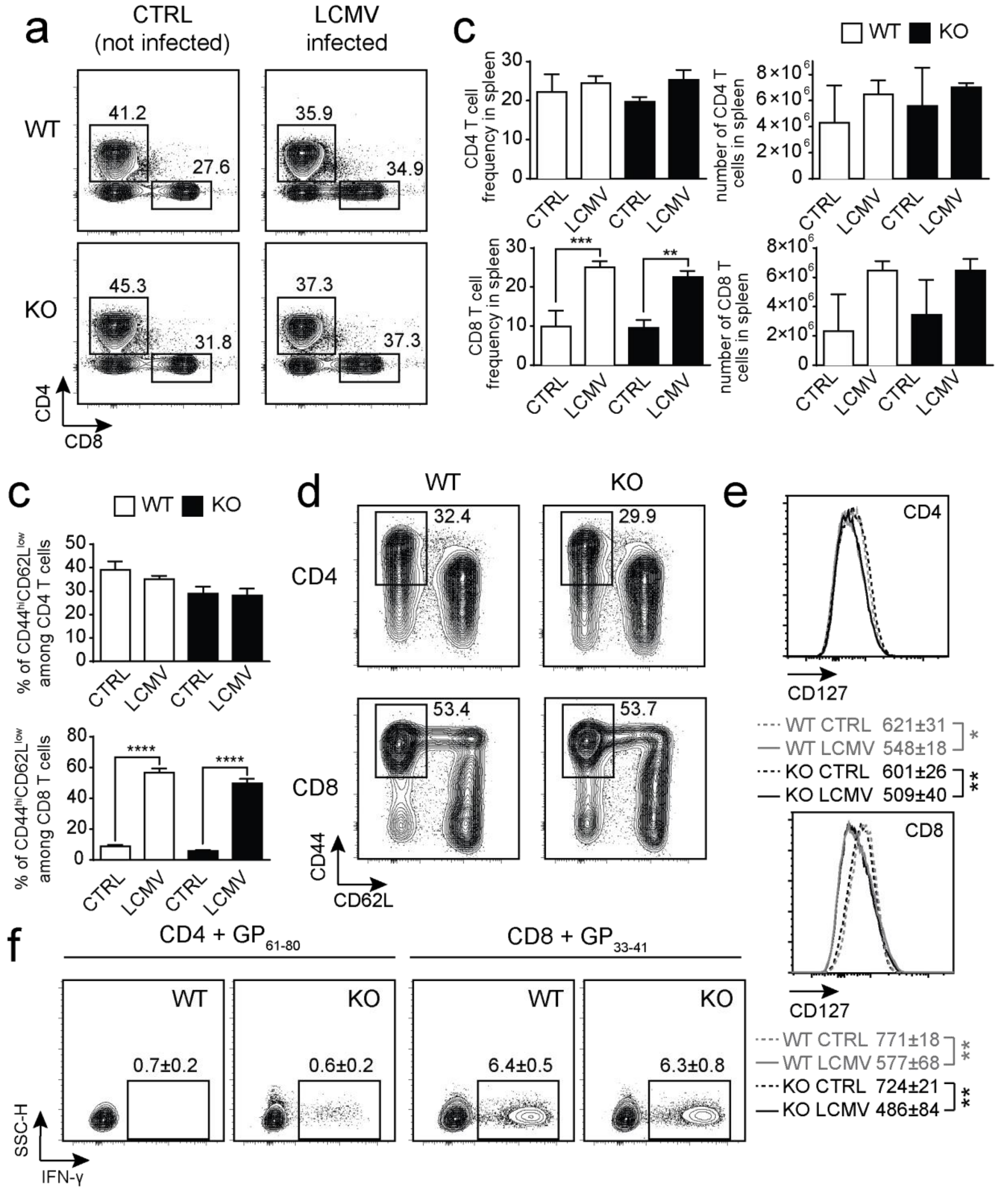

Supplementary figure 9

Supplementary figure 9. Acute LCMV infection affects equally miR-21 WT and KO mice also at a late time point. Armstrong LCMV was injected intravenously at day 0, mice were sacrificed 34 days later and the spleen was analyzed. As a control (CTRL), not infected mice were sacrificed in parallel. **(a)** CD4/CD8 representative stainings, gated on $CD19^{-}MHCIa^{b-}$ cells. **(b)** Summarizing graphs of CD4 and CD8 percentages and absolute numbers. **(c)** Frequencies of $CD44^{hi}CD62L^{low}$ cells among $CD4^{+}$ or $CD8^{+}$ T lymphocytes. **(d)** Representative stainings of CD44 and CD62L in $CD4^{+}$ or $CD8^{+}$ T cells. **(e)** Histograms depicting CD127 expression in $CD4^{+}$ or $CD8^{+}$ T cells. Numbers represent MFI ± SD. **(f)** IFN-γ production in $CD4^{+}$ cells restimulated for 5 h with brefeldin A and $GP_{61–80}$ peptide, as well as $CD8^{+}$ cells with GP_{33-41} peptide, both specific to activate LCMV-restricted T cells. Gates were positioned on empty areas in plots obtained from cells treated with Brefeldin A only. Histograms data represent mean ± SD. One of 2 comparable experiments, each performed with 5–8 mice per group, is shown. ** $P \leq 0.01$, *** $P \leq 0.001$, **** $P \leq 0.0001$ by ANOVA.

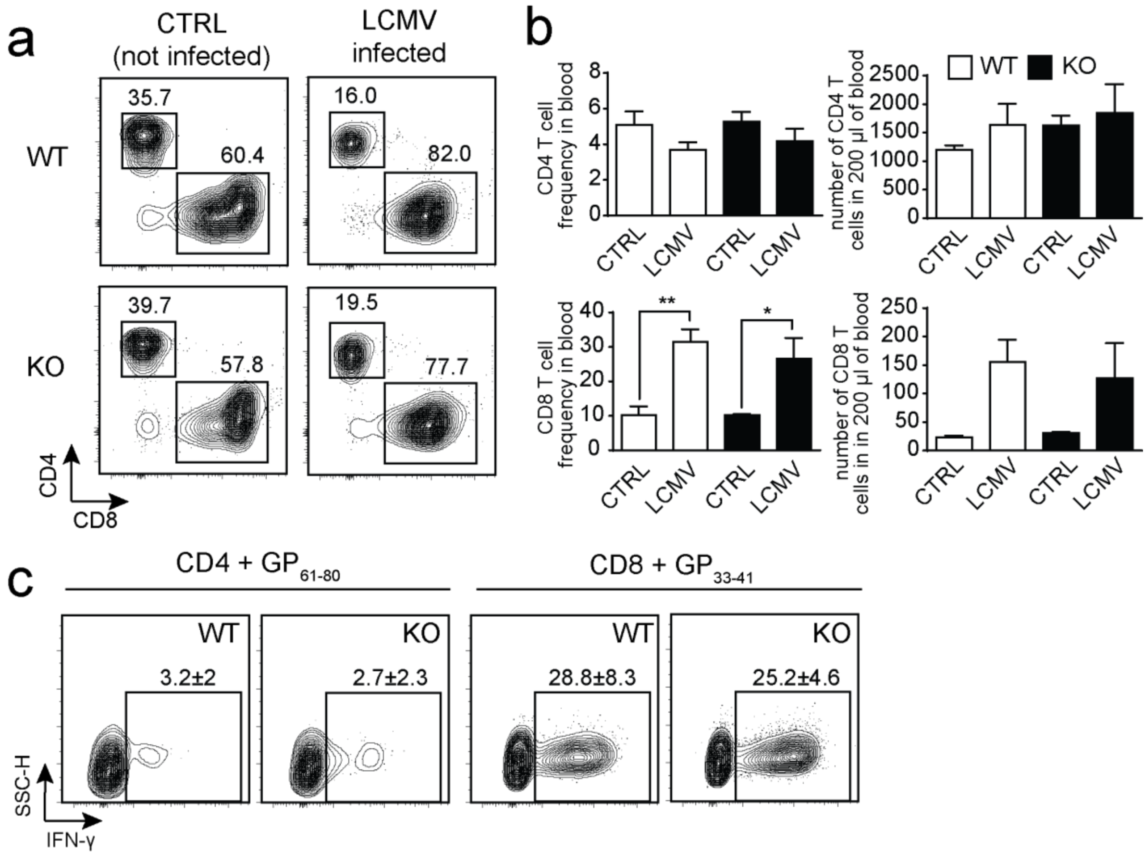

Supplementary figure 10. Chronic LCMV infection affects equally miR-21 WT and KO mice at an early time point during longitudinal analysis. Clone 13 LCMV was injected intravenously at day 0, blood was drawn at day 8 from infected and not infected mice (CTRL), as a control. **(a)** CD4/CD8 representative stainings, gated on CD19$^-$MHCIa^{b-} cells. **(b)** Summarizing graphs of CD4 and CD8 percentages and absolute numbers. **(c)** IFN-γ production in CD4$^+$ cells restimulated for 5 h with brefeldin A and $GP_{61–80}$ peptide, as well as CD8$^+$ cells with GP_{33-41} peptide, both specific to activate LCMV-restricted T cells. Gates were positioned on empty areas in plots obtained from cells treated with Brefeldin A only. Histograms data represent mean ± SD. One of 2 comparable experiments, each performed with 4-7 mice per group, is shown. * $P \leq 0.05$ ** $P \leq 0.01$ by ANOVA.

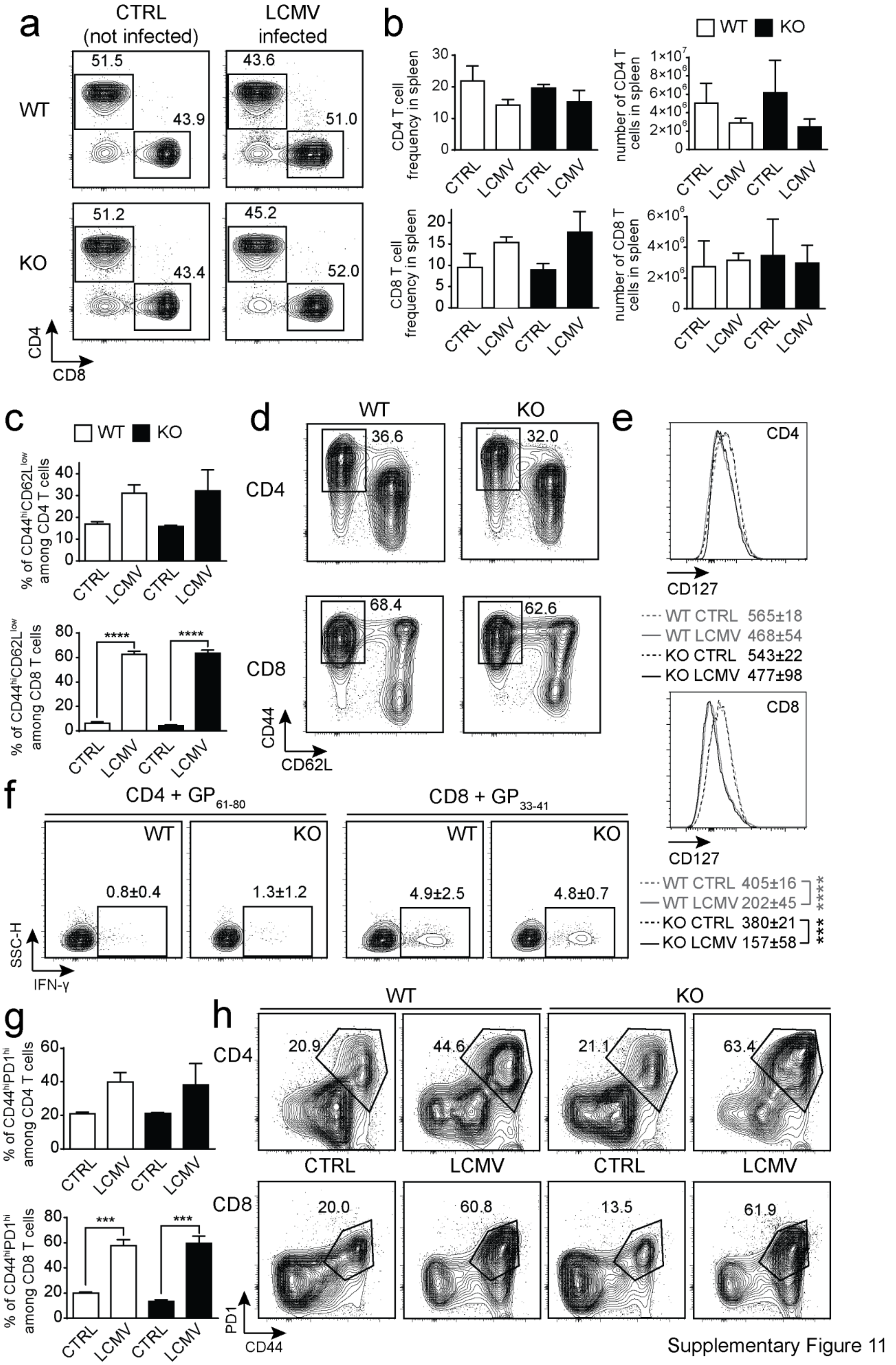

Supplementary Figure 11

Supplementary figure 11. Chronic LCMV infection affects equally miR-21 WT and KO mice. Clone 13 LCMV was injected intravenously at day 0, mice were sacrificed 34 days later and the spleen was analyzed. As a control (CTRL), not infected mice were sacrificed in parallel. **(a)** CD4/CD8 representative stainings, gated on $CD19^{-}MHCIa^{b-}$ cells. **(b)** Summarizing graphs of CD4 and CD8 percentages and absolute numbers. **(c)** Frequencies of $CD44^{hi}CD62L^{low}$ cells among $CD4^{+}$ or $CD8^{+}$ T lymphocytes. **(d)** Representative stainings of CD44 and CD62L in $CD4^{+}$ or $CD8^{+}$ T cells. **(e)** Histograms depicting CD127 expression in $CD4^{+}$ or $CD8^{+}$ T cells. Numbers represent MFI ± SD. **(f)** IFN-γ production in $CD4^{+}$ cells restimulated for 5 h with brefeldin A and $GP_{61–80}$ peptide, as well as $CD8^{+}$ cells with GP_{33-41} peptide, both specific to activate LCMV-restricted T cells. Gates were positioned on empty areas in plots obtained from cells treated with Brefeldin A only. **(g)** Summarizing graphs with the frequencies of $CD44^{hi}PD1^{hi}$ exhausted CD4 and CD8 T cells. **(h)** Representative plots of $CD44^{hi}PD1^{hi}$ exhausted CD4 and CD8 T cells. Histograms data represent mean ± SD. One of 2 comparable experiments, each performed with 4–7 mice per group, is shown. * $P \leq 0.05$ ** $P \leq 0.01$, *** $P \leq 0.001$, **** ^ ≤ 0.0001 by ANOVA.

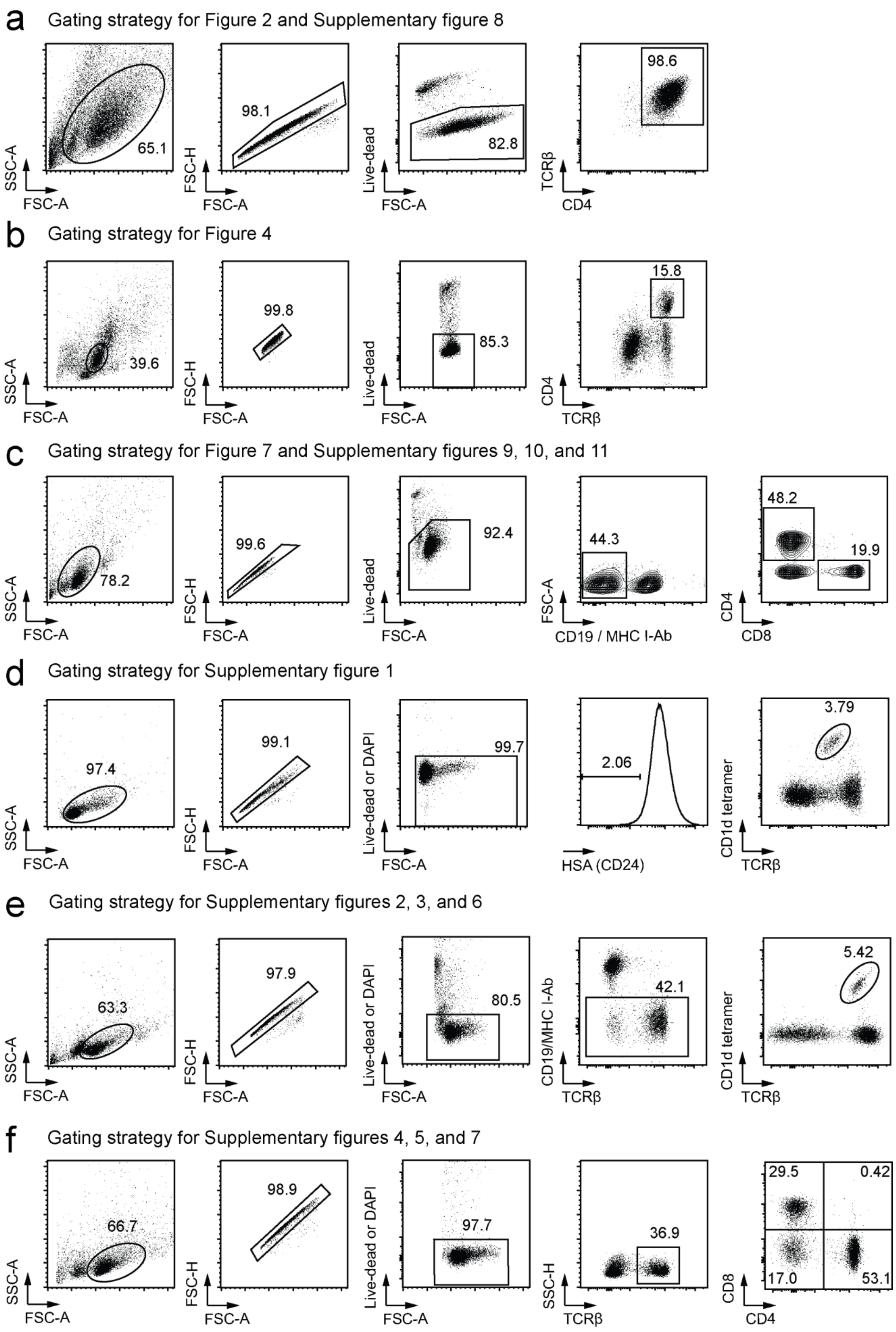

Supplementary Figure 12

Supplementary figure 12. Gating strategies. Depicted are the gating strategies for the cytofluorimetric analyses of: **(a)** Figure 2 and Supplementary figure 8, as lymphocytes, singlets, viable cells, $CD4^{+}TCR\beta^{+}$ cells; **(b)** Figure 4, as lymphocytes, singlets, viable cells, $CD4^{+}TCR\beta^{+}$ cells; **(c)** Figure 7 and Supplementary figures 9, 10 and 11, as lymphocytes, singlets, viable cells, $CD19^{-}MHCIAb^{-}$ cells, $CD4^{+}$ or $CD8^{+}$ cells; **(d)** Supplementary figure 1, as lymphocytes, singlets, viable cells, HSA^{low} cells, iNKT cells; **(e)** Supplementary figures 2, 3 and 6, as lymphocytes, singlets, viable cells, $CD19^{-}MHCIAb^{-}$ cells, iNKT cells; **(f)** Supplementary figures 4, 5, and 7, as lymphocytes, singlets, viable cells, $TCR\beta^{+}$ cells, $CD4^{+}$ or $CD8^{+}$ cells.

Supplementary Tables

Supplementary table 1. Conventional T cells numbers and percentages are correct in miR-21 KO mice. Cells were purified from blood, spleen, LN and liver as described in materials and methods and counted. Lymphocytes were identified by cytofluorimetric analyses through FSC-A vs SSC-A plotting and counts were adjusted accordingly to the identified percentage. TCR-β^+, CD4$^+$ and CD8$^+$ cells were identified through staining with fluorochrome conjugated Ab.

Organ	Data	WT	KO
Blood	number of lymphocytes/μl	4444 ± 1895	4999 ± 447
	% of TCR-β^+ cells among lymphocytes	46.3 ± 4.2	44.6 ± 4.4
	% of CD4$^+$ among TCR-β^+ cells	54.8 ± 2.3	51.8 ± 6.92
	% of CD8$^+$ among TCR-β^+ cells	37.2 ± 2.4	37.6 ± 3.1
Spleen	number of lymphocytes	8.19X10^7 ± 1.64X10^7	6.86X10^7 ± 1.70X10^7
	% of TCR-β^+ cells among lymphocytes	36.1 ± 1.1	33.1 ± 5.5
	% of CD4$^+$ among TCR-β^+ cells	57.7 ± 0.9	53.8 ± 4.8
	% of CD8$^+$ among TCR-β^+ cells	34.7 ± 0.6	36.7 ± 1.9
LN	number of lymphocytes	2.18X10^6 ± 2.35X10^6	3.89X10^5 ± 2.81X10^5
	% of TCR-β^+ cells among lymphocytes	63.9 ± 5.6	61.6 ± 3.7
	% of CD4$^+$ among TCR-β^+ cells	56.0 ± 0.7	54.2 ± 2.5
	% of CD8$^+$ among TCR-β^+ cells	40.2 ± 1.3	41.9 ± 2.6
Liver	number of lymphocytes	3.46X10^6 ± 1.16X10^6	3.58X10^6 ± 9.29X10^5
	% of TCR-β^+ cells among lymphocytes	14.8 ± 1.8	15.1 ± 3.4
	% of CD4$^+$ among TCR-β^+ cells	76.7 ± 4.4	72.6 ± 1.5
	% of CD8$^+$ among TCR-β^+ cells	19.5 ± 3.5	23.1 ± 1.1

Supplementary table 2. Fluctuations in splenocyte counts due to LCMV infection are normal in miR-21 KO mice. Splenocytes counts from LCMV infection experiments shown in Figure 7 and in Supplementary figures 8–10.

		Arm			
LCMV Infection	Day	WT CTRL	WT LCMV	KO CTRL	KO LCMV
Armstrong - acute	8	$4.07\text{x}10^{7} \pm 3.79\text{x}10^{7}$	$7.89\text{x}10^{7} \pm 5.34\text{x}10^{7}$	$4.06\text{x}10^{7} \pm 4.06\text{x}10^{7}$	$1.19\text{x}10^{8} \pm 5.14\text{x}10^{7}$
Armstrong - acute	34	$5.15\text{x}10^{7} \pm 4.49\text{x}106$	$3.28\text{x}10^{7} \pm 7.94\text{x}10^{6}$	$4.80\text{x}10^{7} \pm 2.03\text{x}10^{7}$	$3.47\text{x}10^{7} \pm 7.03\text{x}10^{6}$
Cl.13 - chronic	36	$6.17\text{x}10^{7} \pm 1.33\text{x}10^{7}$	$3.39\text{x}10^{7} \pm 7.87\text{x}10^{6}$	$5.90\text{x}10^{7} \pm 4.22\text{x}10^{7}$	$2.33\text{x}10^{7} \pm 7.07\text{x}10^{6}$
